# Navigating the brain and aging: exploring the impact of transposable elements from health to disease

**DOI:** 10.3389/fcell.2024.1357576

**Published:** 2024-02-27

**Authors:** Anna Le Breton, Margarida P. Bettencourt, Anne-Valerie Gendrel

**Affiliations:** Instituto de Medicina Molecular João Lobo Antunes, Faculdade de Medicina, Universidade de Lisboa, Lisbon, Portugal

**Keywords:** transposable elements, epigenetics, brain, aging, neurological disorders, LINE-1, ERV

## Abstract

Transposable elements (TEs) are mobile genetic elements that constitute on average 45% of mammalian genomes. Their presence and activity in genomes represent a major source of genetic variability. While this is an important driver of genome evolution, TEs can also have deleterious effects on their hosts. A growing number of studies have focused on the role of TEs in the brain, both in physiological and pathological contexts. In the brain, their activity is believed to be important for neuronal plasticity. In neurological and age-related disorders, aberrant activity of TEs may contribute to disease etiology, although this remains unclear. After providing a comprehensive overview of transposable elements and their interactions with the host, this review summarizes the current understanding of TE activity within the brain, during the aging process, and in the context of neurological and age-related conditions.

## Introduction

Transposable elements (TEs) are mobile genetic elements able to move across the genome, independently of their host, either through a cut-and-paste mechanism or by a copy-and-paste mechanism (Wells and Feschotte, 2020). These sequences represent approximately 41% and 48% of the mouse and human genomes respectively, which is particularly relevant when compared to the much smaller percentage of coding sequences (1.5%) (Hermant and Torres-Padilla, 2021; Hoyt et al., 2022). Long considered as purely “junk DNA,” TEs were originally identified in maize by Barbara McClintock more than 60 years ago (McClintock, 1950; McClintock, 1951). She referred to them as “controlling elements” and played a fundamental role in highlighting their capacity to influence gene expression (McClintock, 1956). Since this pioneering work, TEs have been shown to play major roles in genome evolution, structural variation, genome size expansion, spatial organization, genetic diversity and gene regulation (Cordaux and Batzer, 2009; Chuong et al., 2017; Choudhary et al., 2023). On the other hand, unchecked activity of TEs can have nefarious effects, namely inducing mutations, disrupting genes, hindering the transcriptional regulation of genes and leading to the production of extranuclear nucleic acids that can induce cellular toxicity. For that reason, the host maintains a tight control over TE activity, mainly at the transcriptional and epigenetic levels, keeping them silent to prevent deleterious changes. This control is frequently broken in disease, such as cancer and neurological disorders, and during aging. Moreover, silencing mechanisms appear also partially released in certain developmental contexts or tissues, such as in the brain, raising the possibility of an actual functional role conferred by TE activity, in particular in neuronal lineages. However, the contribution of TEs to both physiological and pathological contexts, particularly in the brain, are poorly understood. Specifically, is the aberrant activity of TEs merely a consequence of the disease, or could it contribute to certain pathological phenotypes? In this review, we provide a comprehensive overview about transposable elements and their interactions with the host. Additionally, we summarize the current knowledge regarding TE activity in physiological contexts, with a specific emphasis on the brain and aging, as well as neurological and age-related disorders.

## Insights into transposable elements

### Classification

**FIGURE 1:**
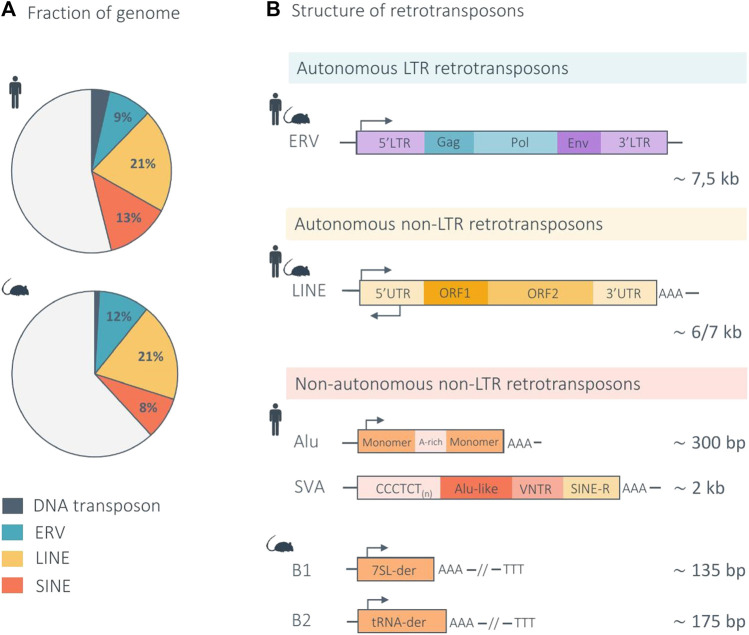
Structure of mammalian retrotransposons and genomic proportions in the human and mouse genome. **(A)** The pie charts indicate the genomic proportion of each retrotransposon class in the human and mouse genome. Light gray represents non-repeat DNA. **(B)** Retrotransposons are divided into two main subclasses according to their mechanism of retrotransposition: LTR and non-LTR retrotransposons. LTR elements, also called endogenous retroviruses (ERVs), are autonomous and share a genomic structure similar in the human and mouse genome. Non-LTR retrotransposons are further divided into two main subtypes: the autonomous LINEs (Long Interspersed Nuclear Elements) and non-autonomous SINEs (Short Interspersed Nuclear Elements). The genomic structure of LINEs is similar in the human and mouse genome. The main non-autonomous non-LTR elements are Alu and SVA in the human genome, and B1 and B2 in the mouse genome. Arrows indicate the approximate position and orientation of the promoter for each element. der, derived (Waterston et al., 2002; Richardson et al., 2015; Deniz et al., 2019; Hoyt et al., 2022).

TEs can be divided into two main classes, according to their transposition mechanism. Class I TEs, or retrotransposons, mobilize their DNA via an RNA intermediate, through a “copy-and-paste” mechanism, in a process known as retrotransposition. Class II TEs, or DNA transposons, mobilize through a “cut-and-paste” mechanism, in a process referred to as transposition (Finnegan, 1989). DNA transposons, which are no longer active in most mammalian species, represent a minority of the human (3%) and mouse (1%) genome (Pace and Feschotte, 2007; Hoyt et al., 2022). In turn, class I retrotransposons constitute the vast majority of TEs in mammals and are divided into two main subclasses according to their mechanism of chromosomal integration: long terminal repeat (LTR) retrotransposons, and non-LTR retrotransposons (Figure 1) (Wells and Feschotte, 2020).

LTR retrotransposons, also called endogenous retroviruses (ERVs), are remnants of exogenous retroviruses that were incorporated in the host germline as a result of ancient viral infections (Mao et al., 2021; Hoyt et al., 2022). A full length, autonomous, ERV has an average length of 7.5 kb and consists of two identical LTRs, which are non-coding regions containing *cis*-regulatory sequences, such as promoters, enhancers, or polyadenylation signals. The LTRs usually flank a set of three ORFs that encode the viral proteins: *gag*, which encodes structural proteins that form virus-like particles (VLPs); *pro*-*pol*, which encodes the enzymes necessary for the viral life cycle (reverse transcriptase, integrase, and protease); and *env*, which encodes the envelope proteins (Figure 1) (Küry et al., 2018). Most ERV copies have accumulated mutations that prevent their retrotransposition. In addition, recombination events between the two LTRs of a proviral insertion often lead to ERVs being reduced to a single LTR, or solo LTR, leaving behind remnants of regulatory sequences scattered throughout the genome (Thomas et al., 2018). All ERV subfamilies are no longer active in the human genome, except the evolutionary young HERV-K subfamily HML-2 (human mouse mammary tumor virus like-2), which shows signs of transcriptional activity and intact ORFs, still capable of producing some of the proteins required for VLPs formation (Garcia-Montojo et al., 2018). In contrast, several ERV subfamilies are still active in mice, such as IAP (intracisternal A-particle) and MusD elements. IAPs are highly abundant and competent for both transcription and retrotransposition. Importantly, ERV insertions contribute to 10%–12% of spontaneous germline mutations in laboratory mice (Maksakova et al., 2006; Stocking and Kozak, 2008).

Non-LTR retrotransposons are composed of two main subtypes: the autonomous LINEs (Long Interspersed Nuclear Elements) and non-autonomous SINEs (Short Interspersed Nuclear Elements).

LINEs constitute approximately 21% of the mammalian genomes and are autonomous, meaning that they produce all the machinery necessary for their own retrotransposition (Fueyo et al., 2022; Hoyt et al., 2022). LINEs are on average 6-7 kb long and composed of a 5′ untranslated region (UTR), comprising an RNA polymerase (RNApol) II promoter with both sense and antisense activity, two open reading frames (ORFs), and a 3′UTR with a polyadenylation signal (Evans and Erwin, 2021). ORF1 encodes an RNA-binding protein that has a nucleic acid chaperone activity required for retrotransposition (Martin and Bushman, 2001; Martin, 2006), while ORF2 encodes a protein with both reverse transcriptase and endonuclease activity (Figure 1) (Mathias et al., 1991; Feng et al., 1996). During retrotransposition, the encoded proteins (ORF1p and ORF2p) bind the RNA from which they originate, in *cis*, and the resulting ribonucleoprotein (RNP) translocates into the nucleus, where reverse transcription and retrotransposition takes place (Terry and Devine, 2020). In mammalian genomes, LINEs are dominated by a single family, LINE-1, which accounts for approximately 17% and 21% of the human and mouse genome respectively, constituting the largest proportion of TE-derived sequences in mammals (Waterston et al., 2002; Terry and Devine, 2020; Hoyt et al., 2022). The majority of LINE-1 copies are no longer functional due to the accumulation of mutations or 5′ truncations. Current estimations predict that only 80–100 LINE-1 copies are intact and still retrotransposition-competent (RC-LINE-1) in humans (Brouha et al., 2003; Evans and Erwin, 2021) and around 3,000 in the mouse genome (DeBerardinis et al., 1998). In addition, a larger number of elements with disrupted ORF sequences still harbor an intact 5′UTR and are hence transcriptionally active (Penzkofer et al., 2017). These elements belong to the evolutionary youngest LINE-1 subfamilies, called L1MdA, L1MdTf and L1MdGf in mice and some of the human-specific LINE-1 (L1Hs) from PA-1 subfamily called the transcribed-active elements subset (L1Ta-subset) in humans (Richardson et al., 2015). It is worth noting that in humans, LINE-1 are the only active autonomous elements (Hoyt et al., 2022).

Unlike LINEs, SINEs are non-autonomous elements. They do not encode any proteins and rely on the machinery produced by LINE-1 elements for their retrotransposition. Although they show a strong *cis*-preference, LINE-1-derived proteins, ORF1p and ORF2p, are able to bind in *trans* SINE RNAs (Dewannieux et al., 2003; Raiz et al., 2012). SINEs are derived from tRNAs or 7SL RNAs (Daniels and Deininger, 1985). Given this ancient origin and due to extensive accumulation of mutations during evolution, current SINE elements are highly diverse. There are two main families of SINEs in the human genome: Alu elements, constituting approximately 11% of the genome and being the TE family with the highest copy number; and the evolutionary young SINE-VNTR-Alu (SVA) elements, comprising only 0.1%–0.2% of the genome (Evans and Erwin, 2021; Hoyt et al., 2022). Alu are approximately 300 bp long, composed of highly similar left and right monomers, transcribed by RNApol III and they terminate with a poly (A) tract (Richardson et al., 2015). SVAs result from the fusion of an Alu sequence, a variable number of tandem repeats (VNTR) and a LTR fragment (SINE-R) (Figure 1). The youngest elements of these families are still active, comprising approximately 200,000 Alu elements from the Y, Ya5, Ya8, and Yb8 subfamilies and around 40% of the youngest SVA elements belonging to SVA-D, SVA-E, SVA-F, and SVA-F1 subfamilies (Comeaux et al., 2009; Hancks and Kazazian, 2010). The main SINE families in mice are the B1 and B2 elements, each representing 2%–3% of the genome (Waterston et al., 2002). If the presence of active SINEs B1 and B2 have been shown by cell culture-based experiments, the exact number of active elements in the mouse genome is still unknown (Dewannieux and Heidmann, 2005).

### Host-transposable element interactions

Although TEs represent a large proportion of mammalian genomes, only a small fraction remains currently active. Indeed, in humans, less than 0.05% of these elements are able to mobilize (Mills et al., 2007). This is because newly inserted TEs usually do not provide an immediate fitness advantage to the host, consequently they tend to become fixed mainly through genetic drift, accumulating neutral mutations over evolutionary time. As a result, older TE insertions in genomes have accumulated mutations that render them non-functional, while more recently inserted TEs retain the capacity for activity, both in terms of transcription and, occasionally, transposition (Bourque et al., 2018). For example, using LINE-1’s allele frequency and sequence divergence as a proxy for age, a study investigated the correlation between LINE-1 activity and age. They found that putative young LINE-1 with low sequence divergence are active in cultured cells and generally polymorphic in the human population. In contrast, highly diverged LINE-1 sequences are most often fixed and inactive (Brouha et al., 2003).

In order to persist throughout evolution, TEs must achieve a delicate equilibrium between their expression and repression in the genome of their hosts. This allows them to replicate and propagate within the genome while avoiding deleterious effects on the host cell functions, as this would not be favorable for their survival (Bourque et al., 2018). The intricate relationship between TEs and the host is thus very complex. A recent review proposed a model to explain host-TE interactions, suggesting that an initial period of cooperation could resolve in one of three ways: conflict or arms race, where the host develops silencing mechanisms to control TEs, which TEs may in turn counteract with anti-silencing mechanisms; cooperation and evasion, where TEs develop self-regulatory mechanisms, which can lead to the development of a mutualistic relationship between TEs and the host; and finally, co-option or domestication, when the host is able to repurpose some of the TE activity for its own benefit (Cosby et al., 2019).

### Molecular and cellular impacts of transposable elements

**FIGURE 2:**
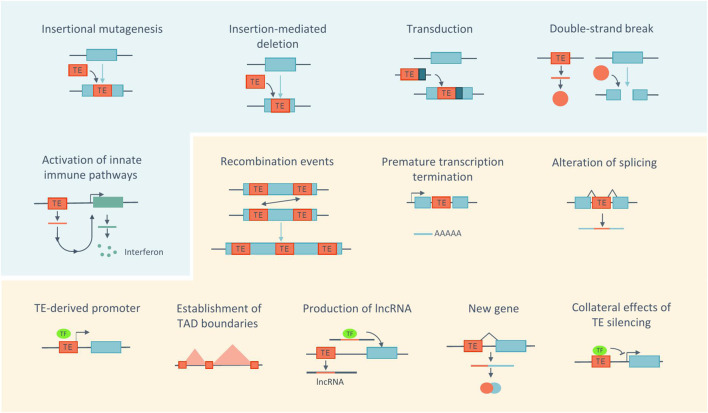
Impact of TEs at the molecular and cellular levels. Actively transposing TEs (blue panel) can be a source of genome instability by inducing mutations, deletions, transduction or DNA damage. The accumulation of products derived from TE may cause inflammation. Even without being active (orange panel), TEs have the ability to alter the host genome through recombination events, alteration of transcription, of gene expression regulation and 3D chromatin architecture. Sequences derived from TEs can also be co-opted by the host. Blue boxes represent genes. TF, transcription factor. Adapted from (Cordaux and Batzer, 2009; Sundaram and Wysocka, 2020; Fueyo et al., 2022).

The presence of TEs can alter the host genome or the transcriptome in numerous ways, contributing to genome evolution and diversification but also potentially affecting genome stability (Figure 2). Active TEs that have the ability to move across the genome represent a source of mutations, as insertions of TEs into protein-coding genes or regulatory regions can disrupt gene function (Cordaux and Batzer, 2009). In addition, insertions can lead to deletions at the target site (Gilbert et al., 2002). TEs may also contribute to exon shuffling through transduction, a process in which flanking sequences are moved with the element and consequently inserted into new locations (Moran et al., 1999; Richardson et al., 2015).

TEs can also contribute to genome instability via their encoded products (Hedges and Deininger, 2007). In particular, the LINE-1 ORF2p encoded-protein can create double-strand breaks (DSBs) at endonuclease target sites (Gasior et al., 2006). In addition, the accumulation of TE-derived products in the cytoplasm, including RNA, extrachromosomal DNA copies and proteins can lead to the activation of innate immune pathways and subsequently trigger inflammation (Saleh et al., 2019).

Furthermore, TEs can induce changes in the host genome even without being active. Recombination events can occur between dispersed TE sequences due to their repetitive nature and high copy number, generating large genomic rearrangements, including deletions, duplications, and inversions (Sen et al., 2006; Han et al., 2008; Lee et al., 2008; Cordaux and Batzer, 2009). In this context, a recent study analyzed the genomes of three individuals and identified 493 genomic rearrangements mediated by TEs, highlighting how this contributes to genome diversity (Balachandran et al., 2022).

In addition, TEs carry a number of regulatory motifs or sequences, which can affect host gene expression, independently of their activity. TEs, such as LINE-1, have internal polyadenylation signals, which can lead to elongation defects, by promoting premature termination of transcription (Perepelitsa-Belancio and Deininger, 2003; Han et al., 2004). TEs, such as Alu and LINE-1, possess splice site signals, which may lead to cryptic splicing, exon skipping or incorporation of their sequence into transcripts (Saleh et al., 2019). Importantly, TEs also contain transcription factor binding sites (TFBSs) and *cis*-regulatory elements (CREs) including promoters and enhancers. The vast majority of them no longer mediate the transcription of TEs, but can be repurposed to regulate the expression of host genes (Sundaram and Wysocka, 2020; Fueyo et al., 2022). An example of this is the transcription of host genes by the LINE-1 antisense promoter in human cells (Nigumann et al., 2002). Additionally, it has been proposed that TEs contribute to the pluripotency gene regulatory network by harboring 25% of the binding sites for pluripotency factors, including OCT4 and NANOG, in both the human and mouse genomes (Kunarso et al., 2010; Sundaram and Wysocka, 2020; Fueyo et al., 2022). Furthermore, different TE families have been shown to contribute to the evolution of innate immunity in mammals by acting as interferon (IFN)-inducible enhancers. In humans, most of these co-opted regulatory elements are found within ERVs (Chuong et al., 2016). However, non-LTR elements, such as L1M2a, were also shown to act as IFN-inducible enhancers (Buttler et al., 2023). In contrast, in mice, B2 elements are the predominant source of these regulatory sequences (Horton et al., 2023).

TEs can also impact host gene expression by affecting the 3D chromatin architecture, either by acting as insulator elements or by being enriched at the boundaries of topologically associating domains (TADs). TADs are chromatin domains where enhancer-promoter interactions are favored, and their boundaries are enriched for binding sites of the zinc finger protein CTCF, many of which derive from TE sequences. This protein not only demarcates these boundaries but also mediates chromatin loop formation (Diehl et al., 2020; Sundaram and Wysocka, 2020; Fueyo et al., 2022).

Beyond regulatory sequences, TE-derived sequences can also be co-opted or exapted for host gene function. One striking example of this are long non-coding RNAs (lncRNAs). Indeed, 83% of the human and 66% of the mouse lncRNAs contain at least one TE (Kelley and Rinn, 2012). The presence of the TE sequence may play a role in regulating lncRNAs expression, processing and localization. For example, they can provide polyadenylation signals or contribute to post-transcriptional adenosine-to-inosine editing. TEs can also serve as functional domains within lncRNAs. Indeed, some studies have demonstrated that mutation or deletion of TEs from the lncRNA sequence can impact its function by altering its localization and expression (Fort et al., 2021).

In addition to lncRNA genes, the exaptation of TE sequences led to the emergence of key protein-coding genes, with both conserved and species-specific functions (Bourque et al., 2018). For example, the coding sequences from different TE families have been domesticated on multiple occasions to integrate genes involved in placental development in both humans and mice (*Syncytin* genes) (Dupressoir et al., 2012) and in brain development (*Arc* gene) (Pastuzyn et al., 2018).

Lastly, TEs can influence, indirectly, the expression of host genes at the epigenetic level as both silencing mechanisms or loss of silencing in certain contexts can spread beyond the TE itself and affect nearby host gene expression (Choi and Lee, 2020; Fueyo et al., 2022).

### Silencing mechanisms

As the immediate uncontrolled activity of TEs can have negative consequences on the genome (see previous section), the host has developed various mechanisms operating at different levels to prevent their expression and transposition (Klein and O’Neill, 2018). At the transcriptional level, silencing mechanisms comprise the deposition of epigenetic modifications on chromatin, primarily involving DNA methylation as well as repressive histone modifications (Garcia-Perez et al., 2016; Deniz et al., 2019; He et al., 2019). Although DNA methylation of CpG-rich promoters is prevalent on most TE families in somatic lineages, these marks are widely erased and reprogrammed during pre-implantation development. Therefore, in embryonic stem cells (ESCs), TEs are primarily repressed through the action of several histone lysine methyltransferases. Hence, in mouse ESCs, trimethylation of lysine 9 on histone H3 (H3K9me3) is deposited by SETDB1 and SUV39H1/2 at specific ERVs and LINE-1 elements, depending on the family considered and their evolutionary age, and is necessary for their silencing and heterochromatinization through HP1 (heterochromatin protein 1) recruitment (Matsui et al., 2010; Karimi et al., 2011; Bulut-Karslioglu et al., 2014). Moreover, dimethylation of lysine 9 on histone H3 (H3K9me2), deposited by the G9a enzyme, is necessary for the silencing of a distinct family of ERVs (MERVL elements) (Maksakova et al., 2013). Repression by trimethylation of H3K27 (H3K27me3) appears limited to a specific ERV family in ESCs (Murine Leukemia Virus (MLV) elements) (Leeb et al., 2010), but can be acquired by other TE families upon genome-wide demethylation (Walter et al., 2016). In parallel, the repressor KAP1 (KRAB-associated protein 1, also called TRIM28) acts as a cofactor essential for silencing and for the recruitment of SETDB1 and other histone-modifying enzymes to specific TE families. KAP1 itself is recruited to TEs by Krüppel-associated box domain-containing zinc finger proteins (KRAB-ZFPs), the largest TF family in mouse and human, which confer the sequence specificity for binding to specific TE families/subfamilies (Schultz et al., 2002) or by the TF Yin Yang 1 (YY1) at specific ERV elements (Lee et al., 2018). KAP1-mediated repression appears particularly relevant for young families of TEs in both mouse and human ESCs. For example, in human ESCs, ancient LINE-1 families have accumulated mutations, rendering them unable to be bound by KAP1/KRAB-ZFPs and to be transcribed. Younger LINE-1 families are bound and repressed by KAP1, while the youngest and more active human-specific L1Hs elements are not yet bound by KAP1/KRAB-ZFPs, but instead repressed by DNA methylation, which may be deposited by small RNA-based mechanisms (Castro-Diaz et al., 2014). Upon implantation and ESC differentiation, permanent silencing of most TEs is ensured by DNA methylation, which is catalyzed by DNA methyltransferases (DNMTs) and then maintained throughout development by the maintenance methyltransferase DNMT1, without the necessity for continual expression of sequence-specific TE-recognizing repressors (Jansz, 2019). DNMTs are recruited to LINE-1 and ERV sequences in ESCs either by the human silencing hub (HUSH) complex, which interacts directly with H3K9me3 or by KAP1/KRAB-ZFPs (Robbez-Masson et al., 2018). Moreover, a binding site for the TF YY1 (Yin Yang 1) located in the 5′UTR and conserved among LINE-1 elements was shown to mediate DNA methylation of young LINE-1 promoters in human ESCs and differentiated cells, possibly through the recruitment of DNMTs (Sanchez-Luque et al., 2019). Furthermore, repression of TEs through KAP1/KRAB-ZFPs, which was initially thought to be restricted to ESCs, is also active in neuronal progenitor cells (NPCs), where KAP1 is necessary for the establishment of H3K9me3 at ERVs and their repression (Fasching et al., 2015; Brattås et al., 2017).

In addition, repression of TEs can also occur at the post-transcriptional level, via RNA silencing-based mechanisms (Heras et al., 2014; Garcia-Perez et al., 2016; Goodier, 2016). A study in human cells showed that the bidirectional transcription of LINE-1 promoters can be processed into small interfering RNAs (siRNAs), which reduce the stability of the LINE-1 RNA (Yang and Kazazian, 2006). In addition, the microRNA miR-128 was shown to inhibit LINE-1 retrotransposition in human induced pluripotent stem cells (iPSCs) and cancer cells, by binding either directly to LINE-1 RNA or to the 3′UTR of nuclear import factor transportin 1 (TNPO1) mRNA, which encodes a protein necessary for the nuclear import of LINE-1 RNP complexes (Hamdorf et al., 2015; Idica et al., 2017). Furthermore, a distinct and conserved pathway active predominantly in germ cells exists, wherein a set of small RNAs called *Piwi*-interacting RNAs (piRNAs) can target complementary TE transcripts for degradation in the cytoplasm and direct DNA methylation to genomic TE sequences (Wang et al., 2023).

Finally, post-translational mediated repression commonly targets the LINE-1 RNP complex for destabilization and degradation (Saleh et al., 2019). It has been proposed that the zinc-finger antiviral protein ZAP colocalizes with LINE-1 RNA and ORF1p in cytoplasmic stress granules to promote RNP degradation, and prevents LINE-1 and Alu retrotransposition (Moldovan and Moran, 2015). Furthermore, uridine residues can be transferred to LINE-1 mRNA in the cytoplasm by TUT7 (terminal uridyl transferase 7) and the MOV10 RNA helicase, which may prevent ORF2p-mediated reverse transcription initiation in the nucleus (Warkocki et al., 2018).

## Transposable elements activity in the healthy brain and during aging

### Healthy brain

Whereas TEs are kept silenced in most somatic tissues, one organ escaping this rule is the brain. Indeed, somatic retrotranspositions have been shown to occur in the healthy human and rodent brain, which could contribute to the establishment of neuronal somatic mosaicism. The first study demonstrating somatic retrotransposition in the neuronal lineage reported mobilization of an engineered human LINE-1 *in vitro,* in NPCs derived from rat hippocampus neural stem cells, and also *in vivo*, in the brain of transgenic mice bearing a similar transgene (Muotri et al., 2005). This was then further shown to occur in NPCs derived from human ESCs or from human fetal brain stem cells (Coufal et al., 2009). In addition, a qPCR assay demonstrated increased endogenous LINE-1 copy number in various brain regions, in particular the hippocampus, compared to the heart and liver of the same donor (Coufal et al., 2009). These observations were confirmed by DNA sequencing approaches, however to different frequencies. Bulk DNA sequencing of various human brain regions identified an extensive number of somatic insertions of LINE-1, as well as Alu and SVA, with widespread events mapping to protein coding genes expressed in the brain (Baillie et al., 2011). Sequencing of single human neuronal nuclei reported frequency of somatic LINE-1 insertions ranging from <0.6 to 13.7 unique insertions per neuron (Evrony et al., 2012; Upton et al., 2015). While the exact rate remains uncertain, collectively, these studies provide evidence of somatic retrotransposition, predominantly impacting LINE-1 elements in the neural lineage, including NPCs and non-dividing neuronal cells (Macia et al., 2017). These observations have important implications for neuronal plasticity and diversity. However, the actual functional significance of these events in brain function remains an open question. Moreover, as recent sequencing studies have focused mainly on somatic retrotransposition events and their frequency, the exact number of individual TE insertions whose expression is perturbed, and the resulting impact on gene regulation through *cis*-regulatory mechanisms, remain unknown.

### Aging

Physiological aging is another process linked with disrupted TE activity. At the molecular level, aging is associated with extensive epigenetic alterations, including changes in histone modifications and DNA methylation patterns, as well as global heterochromatin loss and redistribution (López-Otín et al., 2013). These epigenetic alterations may, in turn, impact the expression and mobilization of TEs in aged cells and tissues (Cardelli, 2018). Changes in chromatin architecture were reported in senescent human fibroblasts, revealing a general compaction of euchromatic gene-rich regions, contrasting with an overall opening of constitutive heterochromatin in gene-poor regions. This was associated with increased expression of evolutionary young subfamilies of Alu, SVA and LINE-1 elements, along with indications of LINE-1 retrotransposition (Cecco et al., 2013a). Similar observations were reported in aged mouse somatic tissues, such as liver and muscle, for various retrotransposon subfamilies (Cecco et al., 2013b). In addition, a progressive increase in TE expression with age was observed in a study that examined total RNA-seq dataset from cell lines derived from healthy individuals from 1 to 94 years-old (LaRocca et al., 2020). At the mechanistic level, besides global epigenetic alterations, it was shown that the binding of sirtuin 6 (SIRT6), a strong repressor of LINE-1 elements, is reduced upon aging. SIRT6 coordinates the packaging of the LINE-1 5′UTR into repressive heterochromatin, through mono-ADP ribosylation of the corepressor KAP1 (Meter et al., 2014).

This raises the question of whether increased transcription and transposition is merely a consequence of aging, or whether it could also actively contribute to it (Maxwell, 2016). Increased transposition could contribute to the elevated DNA damage and related genomic instability associated with aging (Driver and McKechnie, 1992; Laurent et al., 2010; Sedivy et al., 2013). Increased TE expression has also been proposed to actively contribute to aging by promoting sterile inflammation, an aging-associated hallmark (López-Otín et al., 2013). Indeed, WT aged and SIRT6 knockout mice, along with senescent human fibroblasts, present increased expression of LINE-1, as well as elevated cytoplasmic LINE-1 cDNAs. Although the origin of LINE-1 cDNAs in the cytoplasm is still unclear, this can trigger a strong type-I interferon (IFN-I) response, via activation of c-GAS (Cecco et al., 2019; Simon et al., 2019). IFN-I response was mitigated after knockdown of LINE-1 expression using siRNAs, as well as after treatment with nucleoside reverse transcriptase inhibitors (NRTIs), which inhibit the LINE-1 reverse transcriptase (Simon et al., 2019). In addition, in aged mice, NRTI treatment appears to improve age-associated inflammation observed in multiple tissues (Cecco et al., 2019; Simon et al., 2019). Interestingly, healthspan- and lifespan-increasing interventions, such as calorie restriction or pharmacological interventions, were shown to reduce TE expression and LINE-1 transposition in aged mice supporting a potential causal effect of increased TE expression in aging (Cecco et al., 2013b; Wahl et al., 2021). However, diminished TE expression could also be simply a consequence of the reduced aging.

Recently, the HERV-K subfamily HML-2 was also implicated as a potential contributor to cellular senescence through the activation of innate immune pathways. Indeed, it was shown that not only the expression of HML-2 elements is augmented in senescent human mesenchymal stem cells (hMSCs), but also that these elements were able to produce VLPs, which could be released extracellularly and induce senescent phenotypes in young cells (Liu et al., 2023). In addition, increased levels of ERVs were observed in different model organisms, including IAPs and MusD in aged mice (Barbot, 2002; Cecco et al., 2013b), ERV-K and ERV-W in aged cynomolgus monkeys (Liu et al., 2023) and HERV-K, HERV-W and HERV-H in human tissues and serum derived from old individuals (Balestrieri et al., 2015; Nevalainen et al., 2018), as well as in senescent hematopoietic stem cells (Capone et al., 2018). Strikingly, the repression of ERV expression upon treatment with Abacavir, an NRTI, led to an alleviation of cellular senescence and tissue aging in mice (Liu et al., 2023).

Collectively, these findings show that increased expression of TEs is a hallmark of aging. In addition, they pose TEs as key drivers of cellular senescence, primarily through the activation of innate immune pathways, either in a cell-autonomous way or in a paracrine manner in the case of HERV elements. However, questions about the consequences of TEs expression remain to be investigated such as their possible impact on gene expression through *cis*-regulatory mechanism or chromatin architecture modifications. Eventually, TE expression might also be implicated in the development of aging-associated disorders reviewed in the next section.

## Transposable elements activity in neurological and age-related disorders

Active TEs capable of mobilizing in the genome represent a source of genomic variability, which may be harmful to the host. In fact, germline insertions of TEs have been widely linked with genetic diseases (Hancks and Kazazian, 2016). Moreover, somatic *de novo* insertions of TEs have also been reported in various cancers (Burns, 2017). In addition, TEs are able to impact the host even without mobilizing since they bear important regulatory elements and encode proteins with multiple biochemical activities (Wells and Feschotte, 2020). For instance, upon loss of DNA methylation in human NPCs, young LINE-1 elements were shown to function as alternative promoters for various genes with neuronal-related functions or linked to neurological disorders, suggesting that the misregulation of LINE-1 expression during brain development could contribute to the onset of neurological diseases later in life (Jönsson et al., 2019). Accordingly, the misregulation of both the expression and mobilization of TEs has been implicated in several pathological contexts, including in neurological and age-related disorders (Table 1) (Saleh et al., 2019; Ahmadi et al., 2020; Burns, 2020; Terry and Devine, 2020; Evans and Erwin, 2021; Gorbunova et al., 2021). However, in most cases, the contribution of TEs to pathology remains unclear. Therefore, in the following sections, some of the most important findings implicating TEs in different neurological and age-related disease contexts will be discussed.

**TABLE 1:** List of neurological and age-related disorders associated with perturbed TE activity.

Disease	Cause	Implicated TE families	Impact on TE activity and potential TE-driven mechanisms	References
Rett syndrome	Mutation in *MECP2* gene	LINE-1	Increased LINE-1 and IAP expression	Muotri et al. (2010), Skene et al. (2010), Jacob-Hirsch et al. (2018), Zhao et al. (2019)
		ERV	Increased LINE-1 retrotransposition	
Aicardi-Goutières syndrome	Mutations in *TREX1*, *RNASEH2*, *SAMHD1*, *ADAR1* and *IFIH1* genes	LINE-1	Accumulation of DNA and RNA derived from LINE-1 and Alu in the cytosol leading to IFN-1-induced immune response	Crow et al. (2006a), Crow et al. (2006b), Stetson et al. (2008), Zhao et al. (2013), Rice et al. (2014), Hu et al. (2015), Li et al. (2017), Thomas et al. (2017), Benitez-Guijarro et al. (2018), Chung et al. (2018), Herrmann et al. (2018)
		SINE		
Ataxia-telangiectasia	Mutation in *ATM* gene	LINE-1	Increased LINE-1 expression and retrotransposition inducing expression of interferon stimulated genes	Coufal et al. (2011), Jacob-Hirsch et al. (2018), Takahashi et al. (2022)
Amyotrophic lateral sclerosis	TDP-43 cytoplasmic accumulation	ERV	Increased LINE-1, SINE and ERV expression	Li et al. (2015), Liu et al. (2019), Tam et al. (2019)
		LINE-1	Increased LINE-1 retrotransposition	
		SINE	Expression of HERV-K or of its *env* gene leading to neuronal toxicity and cell death	
Frontotemporal dementia	TDP-43 cytoplasmic accumulation	LINE-1	Increased LINE-1, SINE and ERV expression	Li et al. (2012), Liu et al. (2019)
		SINE	Increased LINE-1 retrotransposition	
		ERV		
Alzheimer’s disease	Hyperphosphorylation of Tau protein	LINE-1	Increased expression of LINE-1, SVA, HERV	Guo et al. (2018), Sun et al. (2018), Dembny et al. (2020), Ramirez et al. (2022), Evering et al. (2023)
		SVA	HERV-K(HML-2) RNA activating Toll-like receptors (TLRs)	
		ERV	HERV-K transcripts leading to neurodegeneration and microglia accumulation	
Hutchinson-Gilford Progeria syndrome	Mutation in *LMNA* gene	LINE-1	Increased expression of LINE-1 inhibiting expression of SUV39H1 and inducing heterochromatin loss	Vazquez et al. (2019), LaRocca et al. (2020), Valle et al. (2022), Liu et al. (2023)
		ERV	Increased HERV-K expression and accumulation of VLPs activating innate immune pathways	
Werner syndrome	Mutation in *WRN* gene	LINE-1	Increased expression of LINE-1 inhibiting expression of SUV39H1 and inducing heterochromatin loss	Valle et al. (2022), Liu et al. (2023)
		ERV	Increased HERV-K expression and accumulation of VLPs activating innate immune pathways	

### Rett syndrome

Rett syndrome (RTT) is a neurodevelopmental disorder that affects predominantly young females, with a frequency ranging from 1/10,000 to 1/15,000 live female births (Marano et al., 2021). Clinical features of RTT include regression of spoken language, gait abnormalities and stereotypical hand movements (Neul et al., 2010). RTT symptoms start to manifest in early childhood and develop progressively over stages (Kyle et al., 2018). Approximately 95% of typical RTT cases are caused by mutations in the X-linked methyl-CpG binding protein 2 (*MECP2*) gene (Amir et al., 1999; Neul et al., 2010). *MECP2* encodes an epigenetic regulatory protein, which binds methylated cytosines in CG and CA contexts and interacts with transcriptional co-repressor complexes. As such, one of the main functions of MECP2 is to repress gene expression in a DNA methylation-dependent manner. Moreover, MECP2 is also believed to play a role in transcriptional activation, modulation of alternative splicing and microRNA (miRNA) processing, and chromatin remodeling (Lyst and Bird, 2015; Marano et al., 2021). *MECP2* is ubiquitously expressed, but was shown to be expressed at ∼10-fold higher levels in neurons compared to other cell types, making it one of the most abundant proteins in neuronal nuclei (Skene et al., 2010). Consistent with its high abundance, MECP2 binds methylated DNA broadly throughout the genome and its absence causes global alterations of the neuronal epigenome, leading to transcriptional changes affecting many genes and suggesting that MECP2 fine-tunes neuronal gene expression (Marano et al., 2021).

Besides genes, MECP2 was also shown to bind and repress the expression of methylated TE sequences, such as LINE-1 and IAP retrotransposons in mouse brain (Muotri et al., 2010; Skene et al., 2010). Moreover, increased LINE-1 retrotransposition was observed in neuroepithelial cells of *MECP2*-null mice, in human NPC derived from RTT iPSCs, and in postmortem brains of RTT patients (Muotri et al., 2010). More recently, whole genome sequencing of postmortem brain samples from RTT patients and healthy controls confirmed a higher number of somatic insertions of the human-specific LINE-1 subfamily, L1Hs, in RTT brains (Jacob-Hirsch et al., 2018). In addition, a targeted bulk sequencing approach using PCR revealed that the lack of MECP2 leads to changes in the genomic pattern of L1Hs somatic insertions in cortical neurons of RTT patients. These insertions were found to be enriched in introns and in the sense orientation, which could potentially impact gene expression (Zhao et al., 2019). All together, these studies demonstrated that MECP2 plays a role in the silencing of TE sequences, mainly from the LINE-1 family. However, the extent to which the expression of other TE families is affected in RTT and whether this could play a role in transcriptome changes and in the etiology or progression of RTT remains unknown.

### Aicardi-Goutières syndrome

Aicardi-Goutières syndrome (AGS) is a progressive inflammatory encephalopathy characterized by spasticity, psychomotor retardation, intracranial calcification, white matter changes and cerebrospinal fluid lymphocytosis (Aicardi and Goutières, 1984; Crow et al., 2020; 2013). This syndrome is phenotypically and genotypically heterogeneous, as it can manifest itself with different degrees of severity and results from mutations in various genes involved in nucleic acid metabolism and signaling, including *TREX1*, *RNASEH2*, *SAMHD1*, *ADAR1,* and *IFIH1/MDA5* (Crow et al., 2006a; 2006b; Stephenson, 2008; Rice et al., 2012; 2009; Oda et al., 2014).

The *TREX1* gene encodes the three-prime repair exonuclease 1, an exonuclease involved in the degradation of cytosolic DNA. It was shown that depletion of TREX1 in the mouse leads to the accumulation of single-stranded DNA (ssDNA) derived from TEs, highlighting retroelement-derived DNA as a substrate of TREX1 (Crow et al., 2006a; Stetson et al., 2008). Moreover, an increase in TE derived-extrachromosomal DNA, of which LINE-1 are a major source, was reported in *TREX1*-deficient NPCs obtained after differentiation of human pluripotent stem cells. Further differentiation showed increased apoptosis in neurons and astrocytes exhibiting increased IFN-I secretion, thus contributing to greater neurotoxicity compared to control cells. Knockdown of LINE-1 RNA using shRNAs or inhibition of reverse transcription using NRTIs reduced the levels of extranuclear ssDNA and IFN-1 secretion in *TREX1*-deficient cells (Thomas et al., 2017). It was further shown that TREX1-mediated LINE-1 suppression could also occur independently of its nuclease activity, through ORF1p degradation (Li et al., 2017).

The *RNASEH2A, RNASEH2B* and *RNASEH2C* genes encode the three proteins composing the human ribonuclease H2 enzyme complex. Mutations in any of the three units is the most frequent cause of AGS (Crow et al., 2006b). It has been suggested that RNASEH2 degrades LINE-1 RNA after reverse transcription, being thus required for efficient completion of the retrotransposition cycle. Mutations in the *RNASEH2* genes therefore result in decreased LINE-1 retrotransposition and may lead to the accumulation of cytoplasmic LINE-1 RNA (Benitez-Guijarro et al., 2018).

Mutations in the *SAMHD1* gene also cause AGS (Rice et al., 2009). This gene encodes the SAM domain and HD domain containing protein 1 (SAMHD1), a nucleocytoplasmic shuttling protein with dNTP triphosphohydrolase activity (Du et al., 2019). SAMHD1 is known to inhibit LINE-1 retrotransposition activity in dividing cells by a mechanism still not fully understood (Zhao et al., 2013; Hu et al., 2015; Herrmann et al., 2018). On the one hand, it was suggested that SAMHD1 reduces ORF2p expression (Zhao et al., 2013). On the other hand, SAMHD1 is known to promote the formation of stress granules in the cytoplasm, which may induce the sequestration of LINE-1 RNP and prevent retrotransposition (Hu et al., 2015). The inhibition of LINE-1 retrotransposition by SAMHD1 could restrain TE-derived DNA accumulation in the cytoplasm, preventing the aberrant synthesis of interferon and inflammatory cytokines explaining, at least in part, this characterized feature of AGS associated with SAMHD1 mutations (Hu et al., 2015).

Finally, the *ADAR1* gene encodes an adenosine deaminase acting on double-stranded RNA (dsRNA) (Rice et al., 2012). This protein was shown to bind transcripts derived from Alu elements and to prevent activation of dsRNA sensors such as MDA5, a cytoplasmic viral RNA receptor involved in IFN production and response. *ADAR1* KO in NPCs results in non-edited Alu sequences that tend to form dsRNAs, which trigger IFN-1 production via the activation of MDA5 (Chung et al., 2018). A recent study demonstrated the activation of IFN-1 in the brain of mice carrying *ADAR1* mutation (Guo et al., 2021). In addition, gain-of-function mutations in the *IFIH1* gene, which encodes the MDA5 receptor, were identified in AGS patients. These mutations might lower the recognition threshold of MDA5, enabling not only the recognition of exogenous dsRNA, but also of dsRNA derived from TEs. Eventually, the constitutive activation of the receptor triggers an innate immune response (Rice et al., 2014).

All the mutations described above are associated with the accumulation of cytoplasmic DNA or RNA species derived from TEs. In addition, IFN-1-induced immune response triggering the expression of interferon stimulated genes is observed in almost all AGS patients, except the ones with *RNASEH2B* mutations (Crow and Manel, 2015). However, the mechanistic link between TE product accumulation and the inflammatory phenotype observed in AGS is still poorly understood.

### Ataxia-Telangiectasia

Ataxia-Telangiectasia (AT) is an autosomal recessive disorder characterized by progressive cerebellar degeneration, immunodeficiency, predisposition to develop cancer, radiation sensitivity and premature aging (Rothblum-Oviatt et al., 2016). It is caused by a loss-of-function mutation in the Ataxia-Telangiectasia mutated (*ATM*) gene, which encodes a serine/threonine kinase that activates the DNA repair machinery in response to DNA damage (Savitsky et al., 1995; Shiloh, 2001), highlighting a possible link between genomic instability and neurodegeneration (McKinnon, 2017).

An increase in the retrotransposition efficiency of an engineered human LINE-1 was detected in NPCs derived from *ATM*-deficient hESCs and in *ATM* KO transgenic mice. In addition, an increase in human-specific LINE-1 (L1Hs) copy number was observed in postmortem human brain tissue from AT patients compared to healthy controls (Jacob-Hirsch et al., 2018). This led to the hypothesis that the ATM protein recognizes intermediates created during LINE-1 integration as sites of DNA damage, and consequently increases the number and the length of the resulting retrotransposition events (Coufal et al., 2011).

In a more recent study, an increased expression of evolutionary younger LINE-1 subfamilies and a concomitant decreased expression of TE epigenetic silencers, including MECP2 and KAP1, was observed in cerebellar samples from AT patients. Interestingly, targeted upregulation of the young mouse L1MdTf subfamily using a CRISPR activation (CRISPRa) strategy in the cerebellum of transgenic mice was sufficient to induce progressive ataxia and expression of interferon stimulated genes. In addition, treatment with NRTIs led to the attenuation of disease progression. This study thus delineates a causal link between increased LINE-1 activity and neurodegeneration, using a mouse model (Takahashi et al., 2022). However, whether LINE-1 enhanced activity triggers neurodegeneration directly remains to be functionally demonstrated, as the observed neurodegeneration could be a consequence of increased DNA damage as previously shown (Blaudin de Thé et al., 2018) or of CRISPRa off-target effects.

### Neurodegenerative disorders

In addition to the aforementioned neurological disorders, perturbed TE activity has been linked to various neurodegenerative disorders, some examples of which will be developed in the following section. Amyotrophic lateral sclerosis (ALS), a disease marked by loss of motor neuron function and frontotemporal dementia (FTD), which is associated with loss of frontal and temporal cortexes, are two neurodegenerative diseases associated with disrupted TE activity (Ling et al., 2013). One of the major hallmarks of these pathologies is the loss of nuclear TAR DNA-binding protein 43 (TDP-43) and its subsequent cytoplasmic accumulation (Neumann et al., 2006). This protein was shown to bind to TE-derived transcripts from all the main classes including LINEs, SINEs, and ERVs. The association between TDP-43 and TE-derived transcripts was shown to be reduced in FTD patients and mouse models exhibiting TDP-43 dysfunction show an increase of TE-derived transcripts, which match the ones identified as TDP-43 targets (Li et al., 2012). In agreement with these findings, another study analyzed the transcriptomes of 148 ALS postmortem cortexes and identified a subset of ALS patients with TDP-43 dysfunction and increased expression of TEs, especially young LINE-1 and SVA elements (Tam et al., 2019). Moreover, the effect of nuclear TDP-43 loss and its cytoplasmic aggregation were investigated in postmortem brain samples of FTD and ALS-FTD patients. Chromatin decondensation around LINE-1 insertions was reported, as well as increased LINE-1 DNA content, indicative of increased retrotransposition. Accordingly, it was hypothesized that TDP-43 may regulate the expression of TEs in the brain under physiological conditions through unknown mechanisms, further suggesting that TE derepression may be implicated in ALS and FTD (Liu et al., 2019). TDP-43 was also shown to regulate the expression of HERV-K elements. Indeed, postmortem brain tissue from ALS patients display increased expression of HERV-K. In addition, the expression of HERV-K in human neurons *in vitro* resulted in retraction and beading of neurites, neuronal toxicity and cell death. Moreover, the expression of the HERV-K *env* gene in the neurons of transgenic animals led to the development of several pathological features reminiscent of ALS, including motor dysfunction (Li et al., 2015). All these observations point towards a contribution of HERV-K to neurodegeneration.

Another neurodegenerative disease that has been associated with the activation of TEs is Alzheimer’s disease (AD). One of the neuropathological signatures of AD is the hyperphosphorylation of Tau protein, which leads to the subsequent formation of intracellular neurofibrillary tangles (NFTs) (Iqbal et al., 2005; Klein et al., 2019). Expression of a pathogenic form of Tau was shown to induce heterochromatin loss in motor neurons in mice and in hippocampal neurons from AD patients. The heterochromatin relaxation was shown to be triggered by oxidative stress-induced DNA damage and to be associated with aberrant expression of genes linked with pluripotency and developmental processes, which are normally silent in the brain (Frost et al., 2014). Heterochromatin loss, as well as a reduction of Piwi protein and piRNAs levels, could lead to the increased expression of TEs, including specific subfamilies of LINE-1, SVAs, and HERVs, observed in postmortem brain samples of AD patients. Increased TEs expression could contribute to neurodegeneration by innate immune response activation and/or by promoting genome instability (Guo et al., 2018; Sun et al., 2018).

A recent study investigating TE expression in the brain of three different tauopathy mouse models reported an increase in retrotransposon transcript levels, especially from the ERV class, including IAP, IAP-E, MULV, MERVL, ERV-β4 subfamily members, but also LINE-1 and B1/B2 elements. Moreover, an increase in IAP encoded-gag protein levels and a higher copy number of LINE-1, IAP, ETn and specific ERV-K elements was detected, suggesting that these elements are actively retrotransposing in the context of tauopathy (Ramirez et al., 2022). Moreover, RNA from HERV-K (HML-2) was shown to bind to and activate the murine TLR7 and human TLR8 (Toll-like receptor) expressed in neurons and microglia, resulting in neurodegeneration and microglia accumulation, an important hallmark of AD (Dembny et al., 2020). Recently, a model was proposed to explain the impact of ERV transcripts in neurodegeneration. Innate immune sensors are activated by cytoplasmic HERV-derived nucleic acids, which lead to the secretion of IFN-1 and other inflammatory signals. In response to these signals, microglia release cytokines that can be sensed by astrocytes. These reactive astrocytes produce neurotoxins and are unable to maintain synaptic connections, which could ultimately lead to neuronal death and neurodegeneration (Evering et al., 2023).

### Premature aging progeria syndromes

Another group of age-related disorders where TEs have been implicated are premature aging disorders, including Hutchinson-Gilford Progeria syndrome (HGPS) and Werner syndrome (WS). Both HGPS and WS recapitulate many of the phenotypes associated with normal aging (Ghosh and Zhou, 2014). HGPS is a genetic disorder classified as a laminopathy, caused by single-base substitutions in the *LMNA* encoding lamin A/C, which results in the activation of a cryptic splice site leading to the production of a protein truncated of 50 amino acids, called progerin (Eriksson et al., 2003; Sandre-Giovannoli et al., 2003; Worman and Bonne, 2007; Noda et al., 2015). WS, on the other hand, is caused by mutations in the *WRN* gene, which encodes a RecQ helicase known as the WRN protein that has both exonuclease and helicase activities (Yu et al., 1996; Kudlow et al., 2007). These two premature aging disorders are associated with epigenetic changes, including loss of heterochromatin. Indeed, the lamin A/C proteins, structural components of the nuclear lamina, promote the anchoring of heterochromatin to the nuclear periphery (Goldman et al., 2004; Scaffidi and Misteli, 2005; Shumaker et al., 2006). The WRN protein, known to be involved in DNA repair, plays a role in heterochromatin stability through interactions with heterochromatin proteins, including the histone methyltransferase SUV39H1 and HP1α (Zhang et al., 2015).

Since heterochromatin loss is associated with loss of silencing of TEs, there has been a growing interest in exploring whether TEs could contribute to premature aging disorders. In one study, it was demonstrated that SIRT7-mediated deacetylation of H3K18 plays a role in silencing LINE-1 by facilitating its association with lamin A/C in mouse fibroblasts. Consequently, absence of SIRT7 or depletion of lamin A/C results in transcriptional upregulation of LINE-1 elements in mouse and human cells, consistent with observations from RNA-seq data from fibroblasts of HGPS patients (Vazquez et al., 2019; LaRocca et al., 2020).

Recently, LINE-1 RNA was implicated as a causal agent of heterochromatin erosion in premature aging syndromes. Increased expression of L1Hs elements was observed in hMSCs differentiated from iPSCs derived from HGPS and WS patients. The accumulation of LINE-1 RNA in the nucleus led to an increased interaction with SUV39H1, resulting in the inhibition of its enzymatic activity, loss of heterochromatin and increased expression of senescence-associated secretory phenotype (SASP) genes. Interestingly, an improvement of the senescent phenotype in dermal fibroblasts of progeria patients and HGPS mice was reported following LINE-1 RNA depletion using antisense oligonucleotides (ASOs), but not using NRTIs. In addition, LINE-1 RNA depletion led to an upregulation of pathways associated with nuclear organization, cell proliferation and transcription regulation, together with downregulation of pathways associated with aging, inflammatory response and DNA damage. Together, these results point to an important role of LINE-1 RNAs in the progression of premature aging disorders through the negative regulation of SUV39H1 enzymatic activity (Valle et al., 2022). However, the mechanism by which LINE-1 RNA inhibits SUV39H1 activity remains an open question.

A recent study showed that the HERV-K (HML-2) retrotransposon family also contributes to the senescence phenotype of premature aging syndromes. HERV-K expression was found to be upregulated in HGPS and WS cellular models, where the accumulation of viral proteins and VLPs could trigger innate immune responses thereby contributing to senescence. Importantly, as indicated in the aging section, HERV-K VLPs could be released in a paracrine manner and trigger senescence in non-senescent cells. Consistent with these results, tissues from HGPS cynomolgus monkeys exhibited an increase in ERV-W-Env protein levels. Moreover, this study showed that CRISPRa-mediated activation of HERV-K induced premature senescence, and that repression of HERV-K using shRNA, CRISPR interference or NRTI treatment reduced cellular senescence phenotypes and tissue aging in mice (Liu et al., 2023).

All together, these studies suggest a causal relationship between increased TEs expression and aging-associated phenotypes, which can be alleviated by repressing TEs. This opens up new possibilities for premature aging treatment and offers a strategy to be applied to other aging-associated disorders.

## Concluding remarks

Once considered as purely “junk DNA,” TEs are now recognized as major drivers of genome evolution and genetic diversity. As their immediate impact may be deleterious, the host has developed silencing mechanisms to restrict their expression and retrotransposition, in particular in somatic lineages. It is now accepted that the brain stands out as an exception, exhibiting increased activity of TEs from specific families or subfamilies. It is still unclear whether this is linked to the relaxation of epigenetic mechanisms in neuronal lineages or the presence of specific factors promoting TE expression, or most likely a combination of both. Furthermore, while the biological importance of these observations for neuronal plasticity and diversity is intriguing, it remains unknown and challenging to investigate experimentally.

The aging process, as well as the neurological and age-related disorders described in this review and showing perturbed TE activity, share significant common hallmarks, such as increased DNA damage from retrotransposition, the cytoplasmic accumulation of nucleic acid species from TEs, and the induction of IFN-1 immune response, which can trigger inflammation. Although a causal link between TE expression and neurodegeneration or aging-associated phenotypes is observed in models of AT and progeroid syndromes, the relative contribution of these different features to pathological phenotypes and the sequence of events are unclear. In addition, the potential *cis*-regulatory roles of TE promoters and their influence on transcriptional networks in the various disease contexts remain poorly explored. Regardless, products encoded by TEs, including transcripts and proteins, merit further investigation, in particular as potential candidates for the development of biomarkers of biological age or neurological disorders (LaRocca et al., 2020).

Genome editing technologies such as CRISPR-Cas9 will be essential tools to further unravel the contribution of TEs in physiological or disease contexts (Fueyo et al., 2022). For example, these methods could be used to induce transcriptional silencing of TEs families or subfamilies known to be aberrantly expressed in disease. This would enable determining whether some of the common transcriptome changes or pathological phenotypes are reversed following TE silencing. This could also be used to address whether interfering with TE expression could impact brain development or function.

Furthermore, the development of tools for TE annotation, the more systematic inclusion of TE sequences in next-generation sequencing analysis and the improvement of dedicated computational pipelines will undoubtedly help to understand further the extent to which TE expression and their chromatin state is perturbed in a specific context, as well as the impact on the transcriptome (Lanciano and Cristofari, 2020). In particular, it will be important to distinguish expression of TEs embedded in introns of genes from autonomous expression of TE from their own promoter. In addition, determining whether most elements or only a small subset of insertions from a given family/subfamily are impacted will be essential for the design of downstream functional analysis. Finally, mapping reads coming from the youngest and more active elements, usually overrepresented among the classes showing increased expression in disease (such as L1Hs or HERV-K in the human genome), is very challenging. In that regard, recent pipelines, such as CELLO-seq or SoloTE (Berrens et al., 2022; Rodríguez-Quiroz and Valdebenito-Maturana, 2022), exploit long read and/or single-cell RNA sequencing technologies to tackle many of the issues associated with the mapping of young TEs and allow to analyze more unambiguously TE copies at the individual and locus-specific level.

## References

[B1] Ahmadi A. Toma I. D. Vilor-Tejedor N. Ghamsari M. R. E. Sadeghi I. (2020). Transposable elements in brain health and disease. Ageing Res. Rev. 64, 101153. 10.1016/j.arr.2020.101153 32977057

[B2] Aicardi J. Goutières F. (1984). A Progressive familial encephalopathy in infancy with calcifications of the basal ganglia and chronic cerebrospinal fluid lymphocytosis. Ann. Neurology 15, 49–54. 10.1002/ana.410150109 6712192

[B3] Amir R. E. den Veyver I. B. V. Wan M. Tran C. Q. Francke U. Zoghbi H. Y. (1999). Rett syndrome is caused by mutations in X-linked MECP2, encoding methyl-CpG-binding protein 2. Nat. Genet. 23, 185–188. 10.1038/13810 10508514

[B4] Baillie J. K. Barnett M. W. Upton K. R. Gerhardt D. J. Richmond T. A. Sapio F. D. (2011). Somatic retrotransposition alters the genetic landscape of the human brain. Nature 479, 534–537. 10.1038/nature10531 22037309 PMC3224101

[B5] Balachandran P. Walawalkar I. A. Flores J. I. Dayton J. N. Audano P. A. Beck C. R. (2022). Transposable element-mediated rearrangements are prevalent in human genomes. Nat. Commun. 13 (1), 7115. 10.1038/s41467-022-34810-8 36402840 PMC9675761

[B6] Balestrieri E. Pica F. Matteucci C. Zenobi R. Sorrentino R. Argaw-Denboba A. (2015). Transcriptional activity of human endogenous retroviruses in human peripheral blood mononuclear cells. BioMed Res. Int. 2015, 164529–9. 10.1155/2015/164529 25734056 PMC4334862

[B7] Barbot W. Dupressoir A. Lazar V. Heidmann T. (2002). Epigenetic regulation of an IAP retrotransposon in the aging mouse: progressive demethylation and de-silencing of the element by its repetitive induction. Nucleic Acids Res. 30, 2365–2373. 10.1093/nar/30.11.2365 12034823 PMC117196

[B8] Benitez-Guijarro M. Lopez-Ruiz C. Tarnauskaitė Ž. Murina O. Mohammad M. M. Williams T. C. (2018). RNase H2, mutated in Aicardi-Goutières syndrome, promotes LINE-1 retrotransposition. EMBO J. 37, e98506. 10.15252/embj.201798506 29959219 PMC6068448

[B9] Berrens R. V. Yang A. Laumer C. E. Lun A. T. L. Bieberich F. Law C.-T. (2022). Locus-specific expression of transposable elements in single cells with CELLO-seq. Nat. Biotechnol. 40 (4), 546–554. 10.1038/s41587-021-01093-1 34782740 PMC7614850

[B10] Blaudin de Thé F. Rekaik H. Peze‐Heidsieck E. Massiani-Beaudoin O. Joshi R. L. Fuchs J. (2018). Engrailed homeoprotein blocks degeneration in adult dopaminergic neurons through LINE-1 repression. EMBO J. 37, e97374. 10.15252/embj.201797374 29941661 PMC6068427

[B11] Bourque G. Burns K. H. Gehring M. Gorbunova V. Seluanov A. Hammell M. (2018). Ten things you should know about transposable elements. Genome Biol. 19, 199. 10.1186/s13059-018-1577-z 30454069 PMC6240941

[B12] Brattås P. L. Jönsson M. E. Fasching L. Wahlestedt J. N. Shahsavani M. Falk R. (2017). TRIM28 controls a gene regulatory network based on endogenous retroviruses in human neural progenitor cells. Cell Rep. 18 (1), 1–11. 10.1016/j.celrep.2016.12.010 28052240

[B13] Brouha B. Schustak J. Badge R. M. Lutz-Prigge S. Farley A. H. Moran J. V. (2003). Hot L1s account for the bulk of retrotransposition in the human population. Proc. Natl. Acad. Sci. 100, 5280–5285. 10.1073/pnas.0831042100 12682288 PMC154336

[B14] Bulut-Karslioglu A. De La Rosa-Velázquez I. A. Ramirez F. Barenboim M. Onishi-Seebacher M. Arand J. (2014). Suv39h-Dependent H3K9me3 marks intact retrotransposons and silences LINE elements in mouse embryonic stem cells. Mol. Cell 55, 277–290. 10.1016/j.molcel.2014.05.029 24981170

[B15] Burns K. H. (2017). Transposable elements in cancer. Nat. Rev. Cancer 17, 415–424. 10.1038/nrc.2017.35 28642606

[B16] Burns K. H. (2020). Our conflict with transposable elements and its implications for human disease. Annu. Rev. Pathology Mech. Dis. 15, 51–70. 10.1146/annurev-pathmechdis-012419-032633 31977294

[B17] Buttler C. A. Ramirez D. Dowell R. D. Chuong E. B. (2023). An intronic LINE-1 regulates IFNAR1 expression in human immune cells. Mob. DNA 14 (1), 20. 10.1186/s13100-023-00308-3 38037122 PMC10688052

[B18] Capone S. Connor K. M. Colombo A. Li X. Triche T. J. Ramsingh G. (2018). Senescent human hematopoietic progenitors show elevated expression of transposable elements and inflammatory genes. Exp. Hematol. 62, 33–38. 10.1016/j.exphem.2018.03.003 29549053

[B19] Cardelli M. (2018). The epigenetic alterations of endogenous retroelements in aging. Mech. Ageing Dev. 174, 30–46. 10.1016/j.mad.2018.02.002 29458070

[B20] Castro-Diaz N. Ecco G. Coluccio A. Kapopoulou A. Yazdanpanah B. Friedli M. (2014). Evolutionally dynamic L1 regulation in embryonic stem cells. Genes & Dev. 28, 1397–1409. 10.1101/gad.241661.114 24939876 PMC4083085

[B21] Cecco M. D. Criscione S. W. Peckham E. J. Hillenmeyer S. Hamm E. A. Manivannan J. (2013a). Genomes of replicatively senescent cells undergo global epigenetic changes leading to gene silencing and activation of transposable elements. Aging Cell 12 (2), 247–256. 10.1111/acel.12047 23360310 PMC3618682

[B22] Cecco M. D. Criscione S. W. Peterson A. L. Neretti N. Sedivy J. M. Kreiling J. A. (2013b). Transposable elements become active and mobile in the genomes of aging mammalian somatic tissues. Aging 5, 867–883. 10.18632/aging.100621 24323947 PMC3883704

[B23] Cecco M. D. Ito T. Petrashen A. P. Elias A. E. Skvir N. J. Criscione S. W. (2019). L1 drives IFN in senescent cells and promotes age-associated inflammation. Nature 566, 73–78. 10.1038/s41586-018-0784-9 30728521 PMC6519963

[B24] Choi J. Y. Lee Y. C. G. (2020). Double-edged sword: the evolutionary consequences of the epigenetic silencing of transposable elements. PLOS Genet. 16, e1008872. 10.1371/journal.pgen.1008872 32673310 PMC7365398

[B25] Choudhary M. N. K. Quaid K. Xing X. Schmidt H. Wang T. (2023). Widespread contribution of transposable elements to the rewiring of mammalian 3D genomes. Nat. Commun. 14, 634. 10.1038/s41467-023-36364-9 36746940 PMC9902604

[B26] Chung H. Calis J. J. A. Wu X. Sun T. Yu Y. Sarbanes S. L. (2018). Human ADAR1 prevents endogenous RNA from triggering translational shutdown. Cell 172, 811–824. 10.1016/j.cell.2017.12.038 29395325 PMC5831367

[B27] Chuong E. B. Elde N. C. Feschotte C. (2016). Regulatory evolution of innate immunity through co-option of endogenous retroviruses. Science 351 (6277), 1083–1087. 10.1126/science.aad5497 26941318 PMC4887275

[B28] Chuong E. B. Elde N. C. Feschotte C. (2017). Regulatory activities of transposable elements: from conflicts to benefits. Nat. Rev. Genet. 18, 71–86. 10.1038/nrg.2016.139 27867194 PMC5498291

[B29] Comeaux M. S. Roy-Engel A. M. Hedges D. J. Deininger P. L. (2009). Diverse *cis* factors controlling *Alu* retrotransposition: what causes *Alu* elements to die? Genome Res. 19, 545–555. 10.1101/gr.089789.108 19273617 PMC2665774

[B30] Cordaux R. Batzer M. A. (2009). The impact of retrotransposons on human genome evolution. Nat. Rev. Genet. 10, 691–703. 10.1038/nrg2640 19763152 PMC2884099

[B31] Cosby R. L. Chang N.-C. Feschotte C. (2019). Host–transposon interactions: conflict, cooperation, and cooption. Genes & Dev. 33, 1098–1116. 10.1101/gad.327312.119 31481535 PMC6719617

[B32] Coufal N. G. Garcia-Perez J. L. Peng G. E. Marchetto M. C. N. Muotri A. R. Mu Y. (2011). Ataxia telangiectasia mutated (ATM) modulates long interspersed element-1 (L1) retrotransposition in human neural stem cells. Proc. Natl. Acad. Sci. 108, 20382–20387. 10.1073/pnas.1100273108 22159035 PMC3251057

[B33] Coufal N. G. Garcia-Perez J. L. Peng G. E. Yeo G. W. Mu Y. Lovci M. T. (2009). L1 retrotransposition in human neural progenitor cells. Nature 460, 1127–1131. 10.1038/nature08248 19657334 PMC2909034

[B34] Crow Y. J. Hayward B. E. Parmar R. Robins P. Leitch A. Ali M. (2006a). Mutations in the gene encoding the 3′-5′ DNA exonuclease TREX1 cause Aicardi-Goutières syndrome at the AGS1 locus. Nat. Genet. 38, 917–920. 10.1038/ng1845 16845398

[B35] Crow Y. J. Leitch A. Hayward B. E. Garner A. Parmar R. Griffith E. (2006b). Mutations in genes encoding ribonuclease H2 subunits cause Aicardi-Goutières syndrome and mimic congenital viral brain infection. Nat. Genet. 38, 910–916. 10.1038/ng1842 16845400

[B36] Crow Y. J. Manel N. (2015). Aicardi–Goutières syndrome and the type I interferonopathies. Nat. Rev. Immunol. 15, 429–440. 10.1038/nri3850 26052098

[B37] Crow Y. J. Shetty J. Livingston J. H. (2020). Treatments in aicardi–goutières syndrome. Dev. Med. Child Neurology 62, 42–47. 10.1111/dmcn.14268 31175662

[B38] Crow Y. J. Vanderver A. Orcesi S. Kuijpers T. W. Rice G. I. (2013). Therapies in aicardi–goutières syndrome. Clin. Exp. Immunol. 175, 1–8. 10.1111/cei.12115 PMC3898548 23607857

[B39] Daniels G. R. Deininger P. L. (1985). Repeat sequence families derived from mammalian tRNA genes. Nature 317, 819–822. 10.1038/317819a0 3851163

[B40] DeBerardinis R. J. Goodier J. L. Ostertag E. M. Kazazian H. H. (1998). Rapid amplification of a retrotransposon subfamily is evolving the mouse genome. Nat. Genet. 20, 288–290. 10.1038/3104 9806550

[B41] Dembny P. Newman A. G. Singh M. Hinz M. Szczepek M. Krüger C. (2020). Human endogenous retrovirus HERV-K(HML-2) RNA causes neurodegeneration through Toll-like receptors. JCI Insight 5, e131093. 10.1172/jci.insight.131093 32271161 PMC7205273

[B42] Deniz Ö. Frost J. M. Branco M. R. (2019). Regulation of transposable elements by DNA modifications. Nat. Rev. Genet. 20, 417–431. 10.1038/s41576-019-0106-6 30867571

[B43] Dewannieux M. Esnault C. Heidmann T. (2003). LINE-mediated retrotransposition of marked Alu sequences. Nat. Genet. 35, 41–48. 10.1038/ng1223 12897783

[B44] Dewannieux M. Heidmann T. (2005). L1-mediated retrotransposition of murine B1 and B2 SINEs recapitulated in cultured cells. J. Mol. Biol. 349, 241–247. 10.1016/j.jmb.2005.03.068 15890192

[B45] Diehl A. G. Ouyang N. Boyle A. P. (2020). Transposable elements contribute to cell and species-specific chromatin looping and gene regulation in mammalian genomes. Nat. Commun. 11, 1796. 10.1038/s41467-020-15520-5 32286261 PMC7156512

[B46] Driver C. J. I. McKechnie S. W. (1992). Transposable elements as a factor in the aging of *Drosophila melanogaster* . Ann. N. Y. Acad. Sci. 673, 83–91. 10.1111/j.1749-6632.1992.tb27439.x 1336649

[B47] Du J. Peng Y. Wang S. Hou J. Wang Y. Sun T. (2019). Nucleocytoplasmic shuttling of SAMHD1 is important for LINE-1 suppression. Biochem. Biophysical Res. Commun. 510, 551–557. 10.1016/j.bbrc.2019.02.009 30739781

[B48] Dupressoir A. Lavialle C. Heidmann T. (2012). From ancestral infectious retroviruses to *bona fide* cellular genes: role of the captured syncytins in placentation. Placenta 33, 663–671. 10.1016/j.placenta.2012.05.005 22695103

[B49] Eriksson M. Brown W. T. Gordon L. B. Glynn M. W. Singer J. Scott L. (2003). Recurrent *de novo* point mutations in lamin A cause Hutchinson–Gilford progeria syndrome. Nature 423, 293–298. 10.1038/nature01629 12714972 PMC10540076

[B50] Evans T. A. Erwin J. A. (2021). Retroelement-derived RNA and its role in the brain. Seminars Cell & Dev. Biol. 114, 68–80. 10.1016/j.semcdb.2020.11.001 33229216

[B51] Evering T. H. Marston J. L. Gan L. Nixon D. F. (2023). Transposable elements and Alzheimer’s disease pathogenesis. Trends Neurosci. 46, 170–172. 10.1016/j.tins.2022.12.003 36588011

[B52] Evrony G. D. Cai X. Lee E. Hills L. B. Elhosary P. C. Lehmann H. S. (2012). Single-neuron sequencing analysis of L1 retrotransposition and somatic mutation in the human brain. Cell 151, 483–496. 10.1016/j.cell.2012.09.035 23101622 PMC3567441

[B53] Fasching L. Kapopoulou A. Sachdeva R. Petri R. Jönsson M. E. Männe C. (2015). TRIM28 represses transcription of endogenous retroviruses in neural progenitor cells. Cell Rep. 10 (1), 20–28. 10.1016/j.celrep.2014.12.004 25543143 PMC4434221

[B54] Feng Q. Moran J. V. Kazazian H. H. Boeke J. D. (1996). Human L1 retrotransposon encodes a conserved endonuclease required for retrotransposition. Cell 87, 905–916. 10.1016/S0092-8674(00)81997-2 8945517

[B55] Finnegan D. J. (1989). Eukaryotic transposable elements and genome evolution. Trends Genet. 5, 103–107. 10.1016/0168-9525(89)90039-5 2543105

[B56] Fort V. Khelifi G. Hussein S. M. I. (2021). Long non-coding RNAs and transposable elements: a functional relationship. Biochimica Biophysica Acta (BBA) - Mol. Cell Res. 1868, 118837. 10.1016/j.bbamcr.2020.118837 32882261

[B57] Frost B. Hemberg M. Lewis J. Feany M. B. (2014). Tau promotes neurodegeneration through global chromatin relaxation. Nat. Neurosci. 17, 357–366. 10.1038/nn.3639 24464041 PMC4012297

[B58] Fueyo R. Judd J. Feschotte C. Wysocka J. (2022). Roles of transposable elements in the regulation of mammalian transcription. Nat. Rev. Mol. Cell Biol. 23, 481–497. 10.1038/s41580-022-00457-y 35228718 PMC10470143

[B59] Garcia-Montojo M. Doucet-O’Hare T. Henderson L. Nath A. (2018). Human endogenous retrovirus-K (HML-2): a comprehensive review. Crit. Rev. Microbiol. 44, 715–738. 10.1080/1040841X.2018.1501345 30318978 PMC6342650

[B60] Garcia-Perez J. L. Widmann T. J. Adams I. R. (2016). The impact of transposable elements on mammalian development. Development 143, 4101–4114. 10.1242/dev.132639 27875251 PMC5830075

[B61] Gasior S. L. Wakeman T. P. Xu B. Deininger P. L. (2006). The human LINE-1 retrotransposon creates DNA double-strand breaks. J. Mol. Biol. 357, 1383–1393. 10.1016/j.jmb.2006.01.089 16490214 PMC4136747

[B62] Ghosh S. Zhou Z. (2014). Genetics of aging, progeria and lamin disorders. Curr. Opin. Genet. Dev. 26, 41–46. 10.1016/j.gde.2014.05.003 25005744

[B63] Gilbert N. Lutz-Prigge S. Moran J. V. (2002). Genomic deletions created upon LINE-1 retrotransposition. Cell 110, 315–325. 10.1016/S0092-8674(02)00828-0 12176319

[B64] Goldman R. D. Shumaker D. K. Erdos M. R. Eriksson M. Goldman A. E. Gordon L. B. (2004). Accumulation of mutant lamin A causes progressive changes in nuclear architecture in Hutchinson–Gilford progeria syndrome. Proc. Natl. Acad. Sci. 101, 8963–8968. 10.1073/pnas.0402943101 15184648 PMC428455

[B65] Goodier J. L. (2016). Restricting retrotransposons: a review. Mob. DNA 7, 16. 10.1186/s13100-016-0070-z 27525044 PMC4982230

[B66] Gorbunova V. Seluanov A. Mita P. McKerrow W. Fenyö D. Boeke J. D. (2021). The role of retrotransposable elements in ageing and age-associated diseases. Nature 596, 43–53. 10.1038/s41586-021-03542-y 34349292 PMC8600649

[B67] Guo C. Jeong H.-H. Hsieh Y.-C. Klein H.-U. Bennett D. A. De Jager P. L. (2018). Tau activates transposable elements in Alzheimer’s disease. Cell Rep. 23, 2874–2880. 10.1016/j.celrep.2018.05.004 29874575 PMC6181645

[B68] Guo X. Wiley C. A. Steinman R. A. Sheng Y. Ji B. Wang J. (2021). Aicardi-Goutières syndrome-associated mutation at ADAR1 gene locus activates innate immune response in mouse brain. J. Neuroinflammation 18, 169. 10.1186/s12974-021-02217-9 34332594 PMC8325854

[B69] Hamdorf M. Idica A. Zisoulis D. G. Gamelin L. Martin C. Sanders K. J. (2015). miR-128 represses L1 retrotransposition by binding directly to L1 RNA. Nat. Struct. Mol. Biol. 22, 824–831. 10.1038/nsmb.3090 26367248

[B70] Han J. S. Szak S. T. Boeke J. D. (2004). Transcriptional disruption by the L1 retrotransposon and implications for mammalian transcriptomes. Nature 429, 268–274. 10.1038/nature02536 15152245

[B71] Han K. Lee J. Meyer T. J. Remedios P. Goodwin L. Batzer M. A. (2008). L1 recombination-associated deletions generate human genomic variation. Proc. Natl. Acad. Sci. 105, 19366–19371. 10.1073/pnas.0807866105 19036926 PMC2614767

[B72] Hancks D. C. Kazazian H. H. (2010). SVA retrotransposons: evolution and genetic instability. Seminars Cancer Biol. 20, 234–245. 10.1016/j.semcancer.2010.04.001 PMC2945828 20416380

[B73] Hancks D. C. Kazazian H. H. (2016). Roles for retrotransposon insertions in human disease. Mob. DNA 7, 9. 10.1186/s13100-016-0065-9 27158268 PMC4859970

[B74] He J. Fu X. Zhang M. He F. Li W. Abdul M. M. (2019). Transposable elements are regulated by context-specific patterns of chromatin marks in mouse embryonic stem cells. Nat. Commun. 10 (1), 34. 10.1038/s41467-018-08006-y 30604769 PMC6318327

[B75] Hedges D. J. Deininger P. L. (2007). Inviting instability: transposable elements, double-strand breaks, and the maintenance of genome integrity. Mutat. Research/Fundamental Mol. Mech. Mutagen. 616, 46–59. 10.1016/j.mrfmmm.2006.11.021 PMC1850990 17157332

[B76] Heras S. R. Macias S. Cáceres J. F. Garcia-Perez J. L. (2014). Control of mammalian retrotransposons by cellular RNA processing activities. Mob. Genet. Elem. 4, e28439. 10.4161/mge.28439 PMC4203495 25346866

[B77] Hermant C. Torres-Padilla M.-E. (2021). TFs for TEs: the transcription factor repertoire of mammalian transposable elements. Genes Dev. 35, 22–39. 10.1101/gad.344473.120 33397727 PMC7778262

[B78] Herrmann A. Wittmann S. Thomas D. Shepard C. N. Kim B. Ferreirós N. (2018). The SAMHD1-mediated block of LINE-1 retroelements is regulated by phosphorylation. Mob. DNA 9, 11. 10.1186/s13100-018-0116-5 29610582 PMC5872582

[B79] Horton I. Kelly C. J. Dziulko A. Simpson D. M. Chuong E. B. (2023). Mouse B2 SINE elements function as IFN-inducible enhancers. eLife 12, e82617. 10.7554/eLife.82617 37158599 PMC10229128

[B80] Hoyt S. J. Storer J. M. Hartley G. A. Grady P. G. S. Gershman A. Lima L. G. (2022). From telomere to telomere: the transcriptional and epigenetic state of human repeat elements. Science 376, eabk3112. 10.1126/science.abk3112 35357925 PMC9301658

[B81] Hu S. Li J. Xu F. Mei S. Duff Y. L. Yin L. (2015). SAMHD1 inhibits LINE-1 retrotransposition by promoting stress granule formation. PLOS Genet. 11, e1005367. 10.1371/journal.pgen.1005367 26134849 PMC4489885

[B82] Idica A. Sevrioukov E. A. Zisoulis D. G. Hamdorf M. Daugaard I. Kadandale P. (2017). MicroRNA miR-128 represses LINE-1 (L1) retrotransposition by down-regulating the nuclear import factor TNPO1. J. Biol. Chem. 292, 20494–20508. 10.1074/jbc.M117.807677 28974576 PMC5733588

[B83] Iqbal K. Alonso A. C. Chen S. Chohan M. O. El-Akkad E. Gong C.-X. (2005). Tau pathology in Alzheimer disease and other tauopathies. Biochimica Biophysica Acta (BBA) - Mol. Basis Dis. 1739, 198–210. 10.1016/j.bbadis.2004.09.008 15615638

[B84] Jacob-Hirsch J. Eyal E. Knisbacher B. A. Roth J. Cesarkas K. Dor C. (2018). Whole-genome sequencing reveals principles of brain retrotransposition in neurodevelopmental disorders. Cell Res. 28, 187–203. 10.1038/cr.2018.8 29327725 PMC5799824

[B85] Jansz N. (2019). DNA methylation dynamics at transposable elements in mammals. Essays Biochem. 63, 677–689. 10.1042/EBC20190039 31654072

[B86] Jönsson M. E. Brattås P. L. Gustafsson C. Petri R. Yudovich D. Pircs K. (2019). Activation of neuronal genes via LINE-1 elements upon global DNA demethylation in human neural progenitors. Nat. Commun. 10 (1), 3182. 10.1038/s41467-019-11150-8 31320637 PMC6639357

[B87] Karimi M. M. Goyal P. Maksakova I. A. Bilenky M. Leung D. Tang J. X. (2011). DNA methylation and SETDB1/H3K9me3 regulate predominantly distinct sets of genes, retroelements, and chimeric transcripts in mESCs. Cell Stem Cell 8, 676–687. 10.1016/j.stem.2011.04.004 21624812 PMC3857791

[B88] Kelley D. Rinn J. (2012). Transposable elements reveal a stem cell-specific class of long noncoding RNAs. Genome Biol. 13, R107. 10.1186/gb-2012-13-11-r107 23181609 PMC3580499

[B89] Klein H.-U. McCabe C. Gjoneska E. Sullivan S. E. Kaskow B. J. Tang A. (2019). Epigenome-wide study uncovers large-scale changes in histone acetylation driven by tau pathology in aging and Alzheimer’s human brains. Nat. Neurosci. 22, 37–46. 10.1038/s41593-018-0291-1 30559478 PMC6516529

[B90] Klein S. J. O’Neill R. J. (2018). Transposable elements: genome innovation, chromosome diversity, and centromere conflict. Chromosome Res. 26, 5–23. 10.1007/s10577-017-9569-5 29332159 PMC5857280

[B91] Kudlow B. A. Kennedy B. K. Monnat R. J. (2007). Werner and Hutchinson–Gilford progeria syndromes: mechanistic basis of human progeroid diseases. Nat. Rev. Mol. Cell Biol. 8, 394–404. 10.1038/nrm2161 17450177

[B92] Kunarso G. Chia N.-Y. Jeyakani J. Hwang C. Lu X. Chan Y.-S. (2010). Transposable elements have rewired the core regulatory network of human embryonic stem cells. Nat. Genet. 42, 631–634. 10.1038/ng.600 20526341

[B93] Küry P. Nath A. Créange A. Dolei A. Marche P. Gold J. (2018). Human endogenous retroviruses in neurological diseases. Trends Mol. Med. 24, 379–394. 10.1016/j.molmed.2018.02.007 29551251 PMC7185488

[B94] Kyle S. M. Vashi N. Justice M. J. (2018). Rett syndrome: a neurological disorder with metabolic components. Open Biol. 8, 170216. 10.1098/rsob.170216 29445033 PMC5830535

[B95] Lanciano S. Cristofari G. (2020). Measuring and interpreting transposable element expression. Nat. Rev. Genet. 21, 721–736. 10.1038/s41576-020-0251-y 32576954

[B96] LaRocca T. J. Cavalier A. N. Wahl D. (2020). Repetitive elements as a transcriptomic marker of aging: evidence in multiple datasets and models. Aging Cell 19, e13167. 10.1111/acel.13167 32500641 PMC7412685

[B97] Laurent G. S. Hammell N. McCaffrey T. A. (2010). A LINE-1 component to human aging: do LINE elements exact a longevity cost for evolutionary advantage? Mech. Ageing Dev. 131, 299–305. 10.1016/j.mad.2010.03.008 20346965 PMC2875337

[B98] Lee A. CingÖz O. Sabo Y. Goff S. P. (2018). Characterization of interaction between Trim28 and YY1 in silencing proviral DNA of Moloney murine leukemia virus. Virology 516, 165–175. 10.1016/j.virol.2018.01.012 29407374 PMC8456507

[B99] Lee J. Han K. Meyer T. J. Kim H.-S. Batzer M. A. (2008). Chromosomal inversions between human and chimpanzee lineages caused by retrotransposons. PLoS ONE 3, e4047. 10.1371/journal.pone.0004047 19112500 PMC2603318

[B100] Leeb M. Pasini D. Novatchkova M. Jaritz M. Helin K. Wutz A. (2010). Polycomb complexes act redundantly to repress genomic repeats and genes. Genes & Dev. 24 (3), 265–276. 10.1101/gad.544410 20123906 PMC2811828

[B101] Li P. Du J. Goodier J. L. Hou J. Kang J. Kazazian H. H. (2017). Aicardi–Goutières syndrome protein TREX1 suppresses L1 and maintains genome integrity through exonuclease-independent ORF1p depletion. Nucleic Acids Res. 45, 4619–4631. 10.1093/nar/gkx178 28334850 PMC5416883

[B102] Li W. Jin Y. Prazak L. Hammell M. Dubnau J. (2012). Transposable elements in TDP-43-mediated neurodegenerative disorders. PLoS ONE 7, e44099. 10.1371/journal.pone.0044099 22957047 PMC3434193

[B103] Li W. Lee M.-H. Henderson L. Tyagi R. Bachani M. Steiner J. (2015). Human endogenous retrovirus-K contributes to motor neuron disease. Sci. Transl. Med. 7, 307ra153. 10.1126/scitranslmed.aac8201 PMC6344353 26424568

[B104] Ling S.-C. Polymenidou M. Cleveland D. W. (2013). Converging mechanisms in ALS and FTD: disrupted RNA and protein homeostasis. Neuron 79, 416–438. 10.1016/j.neuron.2013.07.033 23931993 PMC4411085

[B105] Liu E. Y. Russ J. Cali C. P. Phan J. M. Amlie-Wolf A. Lee E. B. (2019). Loss of nuclear TDP-43 is associated with decondensation of LINE retrotransposons. Cell Rep. 27, 1409–1421. 10.1016/j.celrep.2019.04.003 31042469 PMC6508629

[B106] Liu X. Liu Z. Wu Z. Ren J. Fan Y. Sun L. (2023). Resurrection of endogenous retroviruses during aging reinforces senescence. Cell 186, 287–304.e26. 10.1016/j.cell.2022.12.017 36610399

[B107] López-Otín C. Blasco M. A. Partridge L. Serrano M. Kroemer G. (2013). The hallmarks of aging. Cell 153, 1194–1217. 10.1016/j.cell.2013.05.039 23746838 PMC3836174

[B108] Lyst M. J. Bird A. (2015). Rett syndrome: a complex disorder with simple roots. Nat. Rev. Genet. 16, 261–275. 10.1038/nrg3897 25732612

[B109] Macia A. Widmann T. J. Heras S. R. Ayllon V. Sanchez L. Benkaddour-Boumzaouad M. (2017). Engineered LINE-1 retrotransposition in nondividing human neurons. Genome Res. 27, 335–348. 10.1101/gr.206805.116 27965292 PMC5340962

[B110] Maksakova I. A. Romanish M. T. Gagnier L. Dunn C. A. van de Lagemaat L. N. Mager D. L. (2006). Retroviral elements and their hosts: insertional mutagenesis in the mouse germ line. PLoS Genet. 2, e2. 10.1371/journal.pgen.0020002 16440055 PMC1331978

[B111] Maksakova I. A. Thompson P. J. Goyal P. Jones S. J. Singh P. B. Karimi M. M. (2013). Distinct roles of KAP1, HP1 and G9a/GLP in silencing of the two-cell-specific retrotransposon MERVL in mouse ES cells. Epigenetics Chromatin 6, 15. 10.1186/1756-8935-6-15 23735015 PMC3682905

[B112] Mao J. Zhang Q. Cong Y.-S. (2021). Human endogenous retroviruses in development and disease. Comput. Struct. Biotechnol. J. 19, 5978–5986. 10.1016/j.csbj.2021.10.037 34849202 PMC8604659

[B113] Marano D. Fioriniello S. D’Esposito M. Ragione F. D. (2021). Transcriptomic and epigenomic landscape in rett syndrome. Biomolecules 11, 967. 10.3390/biom11070967 34209228 PMC8301932

[B114] Martin S. L. (2006). The ORF1 protein encoded by LINE-1: structure and function during L1 retrotransposition. J. Biomed. Biotechnol. 2006, 45621–45626. 10.1155/JBB/2006/45621 16877816 PMC1510943

[B115] Martin S. L. Bushman F. D. (2001). Nucleic acid chaperone activity of the ORF1 protein from the mouse LINE-1 retrotransposon. Mol. Cell. Biol. 21, 467–475. 10.1128/MCB.21.2.467-475.2001 11134335 PMC86601

[B116] Mathias S. L. Scott A. F. Kazazian H. H. Boeke J. D. Gabriel A. (1991). Reverse transcriptase encoded by a human transposable element. Science 254, 1808–1810. 10.1126/science.1722352 1722352

[B117] Matsui T. Leung D. Miyashita H. Maksakova I. A. Miyachi H. Kimura H. (2010). Proviral silencing in embryonic stem cells requires the histone methyltransferase ESET. Nature 464, 927–931. 10.1038/nature08858 20164836

[B118] Maxwell P. H. (2016). What might retrotransposons teach us about aging? Curr. Genet. 62, 277–282. 10.1007/s00294-015-0538-2 26581630 PMC5120397

[B119] McClintock B. (1950). The origin and behavior of mutable loci in maize. Proc. Natl. Acad. Sci. 36, 344–355. 10.1073/pnas.36.6.344 15430309 PMC1063197

[B120] McClintock B. (1951). Chromosome organization and genic expression. Cold Spring Harb. Symposia Quantitative Biol. 16, 13–47. 10.1101/SQB.1951.016.01.004 14942727

[B121] McClintock B. (1956). Controlling elements and the gene. Cold Spring Harb. Symposia Quantitative Biol. 21, 197–216. 10.1101/SQB.1956.021.01.017 13433592

[B122] McKinnon P. J. (2017). Genome integrity and disease prevention in the nervous system. Genes & Dev. 31, 1180–1194. 10.1101/gad.301325.117 28765160 PMC5558921

[B123] Meter M. V. Kashyap M. Rezazadeh S. Geneva A. J. Morello T. D. Seluanov A. (2014). SIRT6 represses LINE1 retrotransposons by ribosylating KAP1 but this repression fails with stress and age. Nat. Commun. 5, 5011. 10.1038/ncomms6011 25247314 PMC4185372

[B124] Mills R. E. Bennett E. A. Iskow R. C. Devine S. E. (2007). Which transposable elements are active in the human genome? Trends Genet. 23, 183–191. 10.1016/j.tig.2007.02.006 17331616

[B125] Moldovan J. B. Moran J. V. (2015). The zinc-finger antiviral protein ZAP inhibits LINE and Alu retrotransposition. PLOS Genet. 11, e1005121. 10.1371/journal.pgen.1005121 25951186 PMC4423928

[B126] Moran J. V. DeBerardinis R. J. Kazazian H. H. (1999). Exon shuffling by L1 retrotransposition. Science 283, 1530–1534. 10.1126/science.283.5407.1530 10066175

[B127] Muotri A. R. Chu V. T. Marchetto M. C. N. Deng W. Moran J. V. Gage F. H. (2005). Somatic mosaicism in neuronal precursor cells mediated by L1 retrotransposition. Nature 435, 903–910. 10.1038/nature03663 15959507

[B128] Muotri A. R. Marchetto M. C. N. Coufal N. G. Oefner R. Yeo G. Nakashima K. (2010). L1 retrotransposition in neurons is modulated by MeCP2. Nature 468, 443–446. 10.1038/nature09544 21085180 PMC3059197

[B129] Neul J. L. Kaufmann W. E. Glaze D. G. Christodoulou J. Clarke A. J. Bahi-Buisson N. (2010). Rett syndrome: revised diagnostic criteria and nomenclature. Ann. Neurology 68, 944–950. 10.1002/ana.22124 PMC3058521 21154482

[B130] Neumann M. Sampathu D. M. Kwong L. K. Truax A. C. Micsenyi M. C. Chou T. T. (2006). Ubiquitinated TDP-43 in frontotemporal lobar degeneration and amyotrophic lateral sclerosis. Science 314, 130–133. 10.1126/science.1134108 17023659

[B131] Nevalainen T. Autio A. Mishra B. H. Marttila S. Jylhä M. Hurme M. (2018). Aging-associated patterns in the expression of human endogenous retroviruses. PLOS ONE 13, e0207407. 10.1371/journal.pone.0207407 30513106 PMC6279030

[B132] Nigumann P. Redik K. Mätlik K. Speek M. (2002). Many human genes are transcribed from the antisense promoter of L1 retrotransposon. Genomics 79, 628–634. 10.1006/geno.2002.6758 11991712

[B133] Noda A. Mishima S. Hirai Y. Hamasaki K. Landes R. D. Mitani H. (2015). Progerin, the protein responsible for the Hutchinson-Gilford progeria syndrome, increases the unrepaired DNA damages following exposure to ionizing radiation. Genes Environ. 37, 13. 10.1186/s41021-015-0018-4 27350809 PMC4917958

[B134] Oda H. Nakagawa K. Abe J. Awaya T. Funabiki M. Hijikata A. (2014). Aicardi-goutières syndrome is caused by IFIH1 mutations. Am. J. Hum. Genet. 95, 121–125. 10.1016/j.ajhg.2014.06.007 24995871 PMC4085581

[B135] Pace J. K. Feschotte C. (2007). The evolutionary history of human DNA transposons: evidence for intense activity in the primate lineage. Genome Res. 17, 422–432. 10.1101/gr.5826307 17339369 PMC1832089

[B136] Pastuzyn E. D. Day C. E. Kearns R. B. Kyrke-Smith M. Taibi A. V. McCormick J. (2018). The neuronal gene arc encodes a repurposed retrotransposon gag protein that mediates intercellular RNA transfer. Cell 172, 275–288. 10.1016/j.cell.2017.12.024 29328916 PMC5884693

[B137] Penzkofer T. Jäger M. Figlerowicz M. Badge R. Mundlos S. Robinson P. N. (2017). L1Base 2: more retrotransposition-active LINE-1s, more mammalian genomes. Nucleic Acids Res. 45, D68–D73. 10.1093/nar/gkw925 27924012 PMC5210629

[B138] Perepelitsa-Belancio V. Deininger P. (2003). RNA truncation by premature polyadenylation attenuates human mobile element activity. Nat. Genet. 35, 363–366. 10.1038/ng1269 14625551

[B139] Raiz J. Damert A. Chira S. Held U. Klawitter S. Hamdorf M. (2012). The non-autonomous retrotransposon SVA is trans -mobilized by the human LINE-1 protein machinery. Nucleic Acids Res. 40, 1666–1683. 10.1093/nar/gkr863 22053090 PMC3287187

[B140] Ramirez P. Zuniga G. Sun W. Beckmann A. Ochoa E. DeVos S. L. (2022). Pathogenic tau accelerates aging-associated activation of transposable elements in the mouse central nervous system. Prog. Neurobiol. 208, 102181. 10.1016/j.pneurobio.2021.102181 34670118 PMC8712387

[B141] Rice G. I. Bond J. Asipu A. Brunette R. L. Manfield I. W. Carr I. M. (2009). Mutations involved in Aicardi-Goutières syndrome implicate SAMHD1 as regulator of the innate immune response. Nat. Genet. 41, 829–832. 10.1038/ng.373 19525956 PMC4154505

[B142] Rice G. I. Duany Y. T. Jenkinson E. M. Forte G. M. A. Anderson B. H. Ariaudo G. (2014). Gain-of-function mutations in IFIH1 cause a spectrum of human disease phenotypes associated with upregulated type I interferon signaling. Nat. Genet. 46, 503–509. 10.1038/ng.2933 24686847 PMC4004585

[B143] Rice G. I. Kasher P. R. Forte G. M. A. Mannion N. M. Greenwood S. M. Szynkiewicz M. (2012). Mutations in ADAR1 cause Aicardi-Goutières syndrome associated with a type I interferon signature. Nat. Genet. 44, 1243–1248. 10.1038/ng.2414 23001123 PMC4154508

[B144] Richardson S. R. Doucet A. J. Kopera H. C. Moldovan J. B. Garcia-Perez J. L. Moran J. V. (2015). The influence of LINE-1 and SINE retrotransposons on mammalian genomes. Microbiol. Spectr. 3, MDNA3–M0061. 10.1128/microbiolspec.MDNA3-0061-2014 PMC4498412 26104698

[B145] Robbez-Masson L. Tie C. H. C. Conde L. Tunbak H. Husovsky C. Tchasovnikarova I. A. (2018). The HUSH complex cooperates with TRIM28 to repress young retrotransposons and new genes. Genome Res. 28, 836–845. 10.1101/gr.228171.117 29728366 PMC5991525

[B146] Rodríguez-Quiroz R. Valdebenito-Maturana B. (2022). SoloTE for improved analysis of transposable elements in single-cell RNA-Seq data using locus-specific expression. Commun. Biol. 5 (1), 1063. 10.1038/s42003-022-04020-5 36202992 PMC9537157

[B147] Rothblum-Oviatt C. Wright J. Lefton-Greif M. A. McGrath-Morrow S. A. Crawford T. O. Lederman H. M. (2016). Ataxia telangiectasia: a review. Orphanet J. Rare Dis. 11, 159. 10.1186/s13023-016-0543-7 27884168 PMC5123280

[B148] Saleh A. Macia A. Muotri A. R. (2019). Transposable elements, inflammation, and neurological disease. Front. Neurology 10, 894. 10.3389/fneur.2019.00894 PMC6710400 31481926

[B149] Sanchez-Luque F. J. Kempen M.-J. H. C. Gerdes P. Vargas-Landin D. B. Richardson S. R. Troskie R.-L. (2019). LINE-1 evasion of epigenetic repression in humans. Mol. Cell 75, 590–604. 10.1016/j.molcel.2019.05.024 31230816

[B150] Sandre-Giovannoli A. D. Bernard R. Cau P. Navarro C. Amiel J. Boccaccio I. (2003). Lamin A truncation in hutchinson-gilford progeria. Science 300, 2055. 10.1126/science.1084125 12702809

[B151] Savitsky K. Bar-Shira A. Gilad S. Rotman G. Ziv Y. Vanagaite L. (1995). A single ataxia telangiectasia gene with a product similar to PI-3 kinase. Science 268, 1749–1753. 10.1126/science.7792600 7792600

[B152] Scaffidi P. Misteli T. (2005). Reversal of the cellular phenotype in the premature aging disease Hutchinson-Gilford progeria syndrome. Nat. Med. 11, 440–445. 10.1038/nm1204 15750600 PMC1351119

[B153] Schultz D. C. Ayyanathan K. Negorev D. Maul G. G. Rauscher F. J. (2002). SETDB1: a novel KAP-1-associated histone H3, lysine 9-specific methyltransferase that contributes to HP1-mediated silencing of euchromatic genes by KRAB zinc-finger proteins. Genes & Dev. 16, 919–932. 10.1101/gad.973302 11959841 PMC152359

[B154] Sedivy J. M. Kreiling J. A. Neretti N. Cecco M. D. Criscione S. W. Hofmann J. W. (2013). Death by transposition – the enemy within? BioEssays 35, 1035–1043. 10.1002/bies.201300097 24129940 PMC3922893

[B155] Sen S. K. Han K. Wang J. Lee J. Wang H. Callinan P. A. (2006). Human genomic deletions mediated by recombination between Alu elements. Am. J. Hum. Genet. 79, 41–53. 10.1086/504600 16773564 PMC1474114

[B156] Shiloh Y. (2001). ATM and ATR: networking cellular responses to DNA damage. Curr. Opin. Genet. Dev. 11, 71–77. 10.1016/S0959-437X(00)00159-3 11163154

[B157] Shumaker D. K. Dechat T. Kohlmaier A. Adam S. A. Bozovsky M. R. Erdos M. R. (2006). Mutant nuclear lamin A leads to progressive alterations of epigenetic control in premature aging. Proc. Natl. Acad. Sci. 103, 8703–8708. 10.1073/pnas.0602569103 16738054 PMC1472659

[B158] Simon M. Meter M. V. Ablaeva J. Ke Z. Gonzalez R. S. Taguchi T. (2019). LINE1 derepression in aged wild-type and SIRT6-deficient mice drives inflammation. Cell Metab. 29, 871–885.e5. 10.1016/j.cmet.2019.02.014 30853213 PMC6449196

[B159] Skene P. J. Illingworth R. S. Webb S. Kerr A. R. W. James K. D. Turner D. J. (2010). Neuronal MeCP2 is expressed at near histone-octamer levels and globally alters the chromatin state. Mol. Cell 37, 457–468. 10.1016/j.molcel.2010.01.030 20188665 PMC4338610

[B160] Stephenson J. B. P. (2008). Aicardi–goutières syndrome (AGS). Eur. J. Paediatr. Neurology 12, 355–358. 10.1016/j.ejpn.2007.11.010 18343173

[B161] Stetson D. B. Ko J. S. Heidmann T. Medzhitov R. (2008). Trex1 prevents cell-intrinsic initiation of autoimmunity. Cell 134, 587–598. 10.1016/j.cell.2008.06.032 18724932 PMC2626626

[B162] Stocking C. Kozak C. A. (2008). Murine endogenous retroviruses. Cell. Mol. Life Sci. 65, 3383–3398. 10.1007/s00018-008-8497-0 18818872 PMC4802364

[B163] Sun W. Samimi H. Gamez M. Zare H. Frost B. (2018). Pathogenic tau-induced piRNA depletion promotes neuronal death through transposable element dysregulation in neurodegenerative tauopathies. Nat. Neurosci. 21, 1038–1048. 10.1038/s41593-018-0194-1 30038280 PMC6095477

[B164] Sundaram V. Wysocka J. (2020). Transposable elements as a potent source of diverse cis-regulatory sequences in mammalian genomes. Philos. Trans. R. Soc. Lond B Biol. Sci. 375, 20190347. 10.1098/rstb.2019.0347 32075564 PMC7061989

[B165] Takahashi T. Stoiljkovic M. Song E. Gao X.-B. Yasumoto Y. Kudo E. (2022). LINE-1 activation in the cerebellum drives ataxia. Neuron 110, 3278–3287.e8. 10.1016/j.neuron.2022.08.011 36070749 PMC9588660

[B166] Tam O. H. Rozhkov N. V. Shaw R. Kim D. Hubbard I. Fennessey S. (2019). Postmortem cortex samples identify distinct molecular subtypes of ALS: retrotransposon activation, oxidative stress, and activated glia. Cell Rep. 29, 1164–1177.e5. 10.1016/j.celrep.2019.09.066 31665631 PMC6866666

[B167] Terry D. M. Devine S. E. (2020). Aberrantly high levels of somatic LINE-1 expression and retrotransposition in human neurological disorders. Front. Genet. 10, 1244. 10.3389/fgene.2019.01244 31969897 PMC6960195

[B168] Thomas C. A. Tejwani L. Trujillo C. A. Negraes P. D. Herai R. H. Mesci P. (2017). Modeling of TREX1-dependent autoimmune disease using human stem cells highlights L1 accumulation as a source of neuroinflammation. Cell Stem Cell 21, 319–331. 10.1016/j.stem.2017.07.009 28803918 PMC5591075

[B169] Thomas J. Perron H. Feschotte C. (2018). Variation in proviral content among human genomes mediated by LTR recombination. Mob. DNA 9, 36. 10.1186/s13100-018-0142-3 30568734 PMC6298018

[B170] Upton K. R. Gerhardt D. J. Jesuadian J. S. Richardson S. R. Sánchez-Luque F. J. Bodea G. O. (2015). Ubiquitous L1 mosaicism in hippocampal neurons. Cell 161, 228–239. 10.1016/j.cell.2015.03.026 25860606 PMC4398972

[B171] Valle F. D. Reddy P. Yamamoto M. Liu P. Saera-Vila A. Bensaddek D. (2022). LINE-1 RNA causes heterochromatin erosion and is a target for amelioration of senescent phenotypes in progeroid syndromes. Sci. Transl. Med. 14, eabl6057. 10.1126/scitranslmed.abl6057 35947677

[B172] Vazquez B. N. Thackray J. K. Simonet N. G. Chahar S. Kane-Goldsmith N. Newkirk S. J. (2019). SIRT7 mediates L1 elements transcriptional repression and their association with the nuclear lamina. Nucleic Acids Res. 47, 7870–7885. 10.1093/nar/gkz519 31226208 PMC6735864

[B173] Wahl D. Cavalier A. N. Smith M. Seals D. R. LaRocca T. J. (2021). Healthy aging interventions reduce repetitive element transcripts. Journals Gerontology Ser. A 76, 805–810. 10.1093/gerona/glaa302 PMC8087275 33257951

[B174] Walter M. Teissandier A. Pérez-Palacios R. Bourc’his D. (2016). An epigenetic switch ensures transposon repression upon dynamic loss of DNA methylation in embryonic stem cells. eLife 5, e11418. 10.7554/eLife.11418 26814573 PMC4769179

[B175] Wang X. Ramat A. Simonelig M. Liu M.-F. (2023). Emerging roles and functional mechanisms of PIWI-interacting RNAs. Nat. Rev. Mol. Cell Biol. 24, 123–141. 10.1038/s41580-022-00528-0 36104626

[B176] Warkocki Z. Krawczyk P. S. Adamska D. Bijata K. Garcia-Perez J. L. Dziembowski A. (2018). Uridylation by TUT4/7 restricts retrotransposition of human LINE-1s. Cell 174, 1537–1548. 10.1016/j.cell.2018.07.022 30122351 PMC6191937

[B177] Waterston R. H. Lindblad-Toh K. Birney E. Rogers J. Abril J. F. (2002). Initial sequencing and comparative analysis of the mouse genome. Nature 420, 520–562. 10.1038/nature01262 12466850

[B178] Wells J. N. Feschotte C. (2020). A field guide to eukaryotic transposable elements. Annu. Rev. Genet. 54, 539–561. 10.1146/annurev-genet-040620-022145 32955944 PMC8293684

[B179] Worman H. J. Bonne G. (2007). “Laminopathies”: a wide spectrum of human diseases. Exp. Cell Res. 313, 2121–2133. 10.1016/j.yexcr.2007.03.028 17467691 PMC2964355

[B180] Yang N. Kazazian H. H. (2006). L1 retrotransposition is suppressed by endogenously encoded small interfering RNAs in human cultured cells. Nat. Struct. Mol. Biol. 13, 763–771. 10.1038/nsmb1141 16936727

[B181] Yu C.-E. Oshima J. Fu Y.-H. Wijsman E. M. Hisama F. Alisch R. (1996). Positional cloning of the werner’s syndrome gene. Science 272, 258–262. 10.1126/science.272.5259.258 8602509

[B182] Zhang W. Li J. Suzuki K. Qu J. Wang P. Zhou J. (2015). Aging stem cells. A Werner syndrome stem cell model unveils heterochromatin alterations as a driver of human aging. Science 348, 1160–1163. 10.1126/science.aaa1356 25931448 PMC4494668

[B183] Zhao B. Wu Q. Ye A. Y. Guo J. Zheng X. Yang X. (2019). Somatic LINE-1 retrotransposition in cortical neurons and non-brain tissues of Rett patients and healthy individuals. PLOS Genet. 15, e1008043. 10.1371/journal.pgen.1008043 30973874 PMC6478352

[B184] Zhao K. Du J. Han X. Goodier J. L. Li P. Zhou X. (2013). Modulation of LINE-1 and Alu/SVA retrotransposition by aicardi-goutières syndrome-related SAMHD1. Cell Rep. 4, 1108–1115. 10.1016/j.celrep.2013.08.019 24035396 PMC3988314

